# The senescent methylome and its relationship with cancer, ageing and germline genetic variation in humans

**DOI:** 10.1186/s13059-015-0748-4

**Published:** 2015-09-17

**Authors:** Robert Lowe, Marita G. Overhoff, Sreeram V. Ramagopalan, James C. Garbe, James Koh, Martha R. Stampfer, David H. Beach, Vardhman K. Rakyan, Cleo L. Bishop

**Affiliations:** The Blizard Institute, Barts and The London School of Medicine and Dentistry, Queen Mary University of London, 4 Newark Street, London, E1 2AT UK; Life Science Division, Lawrence Berkeley National Laboratory, Berkeley, CA 94720 USA; Division of Surgical Sciences, Department of Surgery, Duke University Medical School, Durham, NC 27710 USA

## Abstract

**Background:**

Cellular senescence is a stable arrest of proliferation and is considered a key component of processes associated with carcinogenesis and other ageing-related phenotypes. Here, we perform methylome analysis of actively dividing and deeply senescent normal human epithelial cells.

**Results:**

We identify senescence-associated differentially methylated positions (senDMPs) from multiple experiments using cells from one donor. We find that human senDMP epigenetic signatures are positively and significantly correlated with both cancer and ageing-associated methylation dynamics. We also identify germline genetic variants, including those associated with the p16INK4A locus, which are associated with the presence of *in vivo* senDMP signatures. Importantly, we also demonstrate that a single senDMP signature can be effectively reversed in a newly-developed protocol of transient senescence reversal.

**Conclusions:**

The senDMP signature has significant potential for understanding some of the key (epi)genetic etiological factors that may lead to cancer and age-related diseases in humans.

**Electronic supplementary material:**

The online version of this article (doi:10.1186/s13059-015-0748-4) contains supplementary material, which is available to authorized users.

## Background

**Fig. 1:**
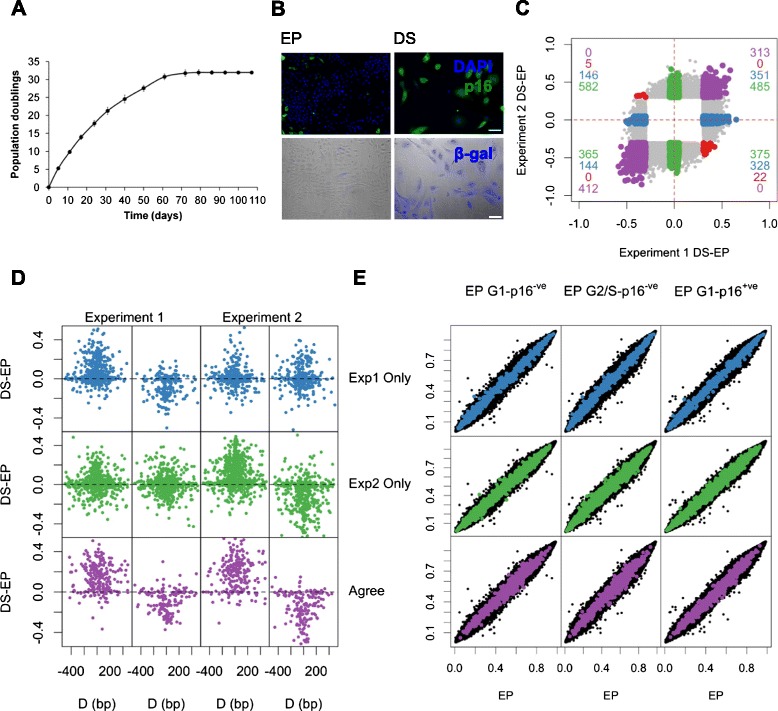
Genome-wide DNA methylation dynamics during cellular senescence. **a** HMECs progressively cease proliferation with serial passage. Early proliferating (EP) and deeply senescent (DS) cells as used throughout are indicated. Error bars = SD of three independent experiments. **b** DS cells display increased p16 expression (green, top row) and SA-β-gal staining (blue, bottom row). **c** A scatter plot of the average beta value differences between triplicate samples of DS and EP cultures for those sites found significantly differentially methylated (F-test; genome wide corrected *P* value <0.01 and a minimum beta value difference of 0.3 between EP and DS cells) in either experiment 1 or experiment 2 (grey points). Those sites which are senDMPs in experiment 1 which show a beta value difference <0.1 in experiment 2 (926 probes, ‘experiment 1’) are highlighted in blue; those senDMPs in experiment 2 which show a beta value difference <0.1 in experiment 1 (1,807 probes, ‘experiment 2 only’:) are highlighted in red and those common senDMPs between experiment 1 and 2 which change in the same direction (725 probes, ‘agree’) are highlighted in green. Those senDMPs in common between experiments 1 and 2 which show opposite directions are highlighted in red. The number of sites for each quadrant are shown in the four corners with the appropriate colour. **d** DS–EP beta value differences of neighbouring Illumina 450K probes within 500 bp of ‘index’ senDMP. The hypermethylated index senDMPs (left) and hypomethylated index senDMPs (right) are shown for both experiment 1 and experiment 2. Each panel row represents the three different sites (experiment 1 only (blue), experiment 2 only (green) and agree (purple)). **e** Comparison of DNA methylation profiles between unsorted EP cells versus each of the three different EP sub-fractionated populations (left, middle and right panels). The beta values for all 401,915 CpGs are plotted on each axis (black points). senDMPs, as defined in the text, are illustrated in blue (experiment 1 only), green (experiment 2 only) and purple (agree). No genome-wide statistical significant differences were found between EP and any of the sub-fractionated populations, or indeed between any of the FACs sorted populations (Additional file 5: Table S8)

Primary human cells display senescence during prolonged propagation *in vitro* [1]. Thus, a culture that might initially multiply with great rapidity eventually slows and reaches a state of replicative exhaustion, or deep senescence, during which viability can be retained from weeks to years (Fig. 1a, Additional file 1: Figure S1). The cell cycle inhibitor p16^INK4A^ (p16) is hallmark of senescence both *in vitro* and *in vivo* [2]. There has been considerable interest in establishing the potential role of cellular senescence in ageing and the many diseases for which age is the primary risk factor [3]. In particular, the pathways that enforce cellular senescence *in vitro*, and in which mutation leads to increased lifespan or even immortality, are invariably disrupted either through epigenetic silencing or mutation, in cancer [4]. This includes, but is not limited to, the p16 pathway. However, it is not always possible to relate *in vitro* cellular senescence, in its various manifestations with potentially related phenomena in ageing or disease.

Methylation of DNA is an epigenetic modification essential for the regulation of mammalian genome function [5]. Patterns of DNA methylation are grossly perturbed in every cancer studied to date [6], and have also been established as a highly effective biomarker of age in humans [7]. DNA methylation thus represents a potentially useful candidate for genome-scale characterisation of senescence. Here, we describe and characterise genome-wide methylation dynamics during cellular senescence, and identify senescence signatures that potentially unite tissue culture senescence with the biology of cancer, and other ageing-related phenotypes. We also find that senDMP signatures can arise *in vivo* as a result of germline genetic variation. Finally, we show that the senescent phenotype and the associated senDMP signatures can be reversed.

## Results

### Cellular senescence is associated with extensive DNA methylome dynamics

Normal human mammary epithelial cells (HMECs) are known to undergo p16-mediated cellular senescence, independent of telomere attrition [8, 9]. Here, HMECs from a healthy 21-year-old female donor were cultured from passage 6 (termed early proliferating (EP)), to deep senescence (DS) (Fig. 1a, Methods). The DS cells in our experiments displayed the key characteristics of cellular senescence, including elevated expression of p16INK4A (p16), a key mediator of the phenotype in these cells, senescence-associated β-galactosidase (SA-β-gal) (Fig. 1b) [9], majority G1 DNA content (data not shown), and no expansion upon at least two further serial passages (Fig. 1a).

To investigate senescence-associated genome-scale DNA methylation dynamics we used Illumina 450K arrays, which assess methylation at >450,000 different cytosine residues associated with the majority of promoters (up to 1.5 kb upstream of the transcriptional start site), CpG islands (CGIs), gene bodies and a variety of intergenic sites including many enhancers [10]. We profiled two separate experiments, termed experiment 1 and experiment 2, which were performed 1 year apart under the same conditions (using the same donor) with the exception of medium/supplement lot numbers. For each experiment three independent EP cultures were serially passaged until they reached DS. As a control for subsequent experiments, EP and DS cultures were exposed to a siRNA termed ‘siGLO’ (targeting *Cyclophilin B*). siGLO did not perturb cellular phenotype (Additional file 1: Figure S2).

DNA methylation profiles revealed excellent correlation among triplicate samples of EP and DS cultures within each experiment (R^2^ >0.99, Additional file 1: Figures S3 and S4). To compare the EP and DS samples across experiments we called differences between experiment 1 and experiment 2 EP cells, finding 872 probes (FDR 1 % (F-test), beta value difference >0.3), and between experiment 1 and experiment 2 DS cells, finding 11,568 probes (FDR 1 % (F-test), beta value difference >0.3). This suggests that the EP methylation state is much more consistent across different experiments than that of the DS methylation state (Additional file 1: Figure S5). To investigate the cause of these differences between DS cells between experiments we subdivided the 11,568 probes into four categories (Methods). Of these, 777 (7 %) probes are associated with an unknown batch effect between the experiments, 6,390 probes (55 %) are associated with a senescent associated change in one experiment and not the other, 4,087 (35 %) probes are associated with a senescent associated change but in the opposite direction between the two experiments and 314 (3 %) show similar directional differences. Therefore the majority of these methylation differences in DS between experiments are due to the different dynamics of senescence.

Next, we identified senescence-associated differentially methylated positions (senDMPs) in DS relative to EP cells, using an F-test (FDR 1 %) and a minimum average beta value difference of 0.3 (Methods). This analysis uncovered 3,852 distinct senDMPs for experiment 1 (2,240 hypermethylated and 1,612 hypomethylated) and 8,158 senDMPs for experiment 2 (3,928 hypermethylated and 4,230 hypomethylated). Seven hundred and fifty-two probes were found to overlap between experiments 1 and 2, representing a highly significant enrichment of probes in common across the two experiments (9.00-fold enrichment; *P* value <10^−4^). Despite this high enrichment this still represents only a 20 % overlap and hence why the DS state is less consistent than that of EP. To investigate this further, we defined three separate senDMP groups; ‘experiment 1 only’: senDMPs in experiment 1 which show a beta value difference <0.1 in experiment 2 (969 probes, blue, Additional file 2: Table S1); ‘experiment 2 only’: senDMPs in experiment 2 which show a beta value difference <0.1 in experiment 1 (1,807 probes, green, Additional file 3: Table S2); and ‘agree’: common senDMPs between experiment 1 and 2 which change in the same direction (725 probes, purple; Fig. 1c, Additional file 4: Table S3). senDMPs in all three groups showed similar dynamics at neighbouring Illumina 450K probes within 500 bp of the ‘index’ CpG suggesting that they belonged to larger regions of differential methylation (ρ = 0.38 (experiment 1 only), ρ = 0.51 (experiment 2 only), ρ = 0.58 (agree), *P* value <2.2×10^−16^ (for all), Fig. 1d, Methods [11]). This suggests that agree sites show similar regional dynamics to that of experiment 1 or experiment 2 only sites, for example, agree sites are no more likely to be found in regions than the others. Examination of various genomic properties yielded only modest fold enrichments across the different senDMP groups (Additional file 1: Figure S6).

Senescence (DS cells) is associated with a G1 cell cycle arrest, whereas all phases of the cell cycle are present during active proliferation (EP cells) (Additional file 1: Figure S7). It was therefore possible that apparent senDMPs might simply reflect different fractional percentages in each cell cycle phase between DS and EP cultures, a key unaddressed issue in previous reports [12, 13]. Thus, we fractionated EP cells generated in experiment 1 into three sub-populations (G1-p16^-ve^, G2/S-p16^-ve^ and G1-p16^+ve^) by flow cytometry (Methods), and analysed each in biological triplicate by Illumina 450K arrays (Additional file 1: Figure S7, Methods). By contrast with the well-described changes in gene expression during the cell cycle, no significant methylation differences were observed between unsorted EP cells and any of the three EP sub-populations, including the EP cells that express p16 (G1-p16^+ve^ cells) (all R^2^ >0.99, Fig. 1e, Additional file 5: Table S8). These data suggest that the senDMP signature is a product of deep senescence and not simply p16 status. It also suggests that within the cell populations used in these experiments there is no evidence of a regulation of the cell cycle by DNA methylation-associated changes.

### Reversal of senescence

**Fig. 2:**
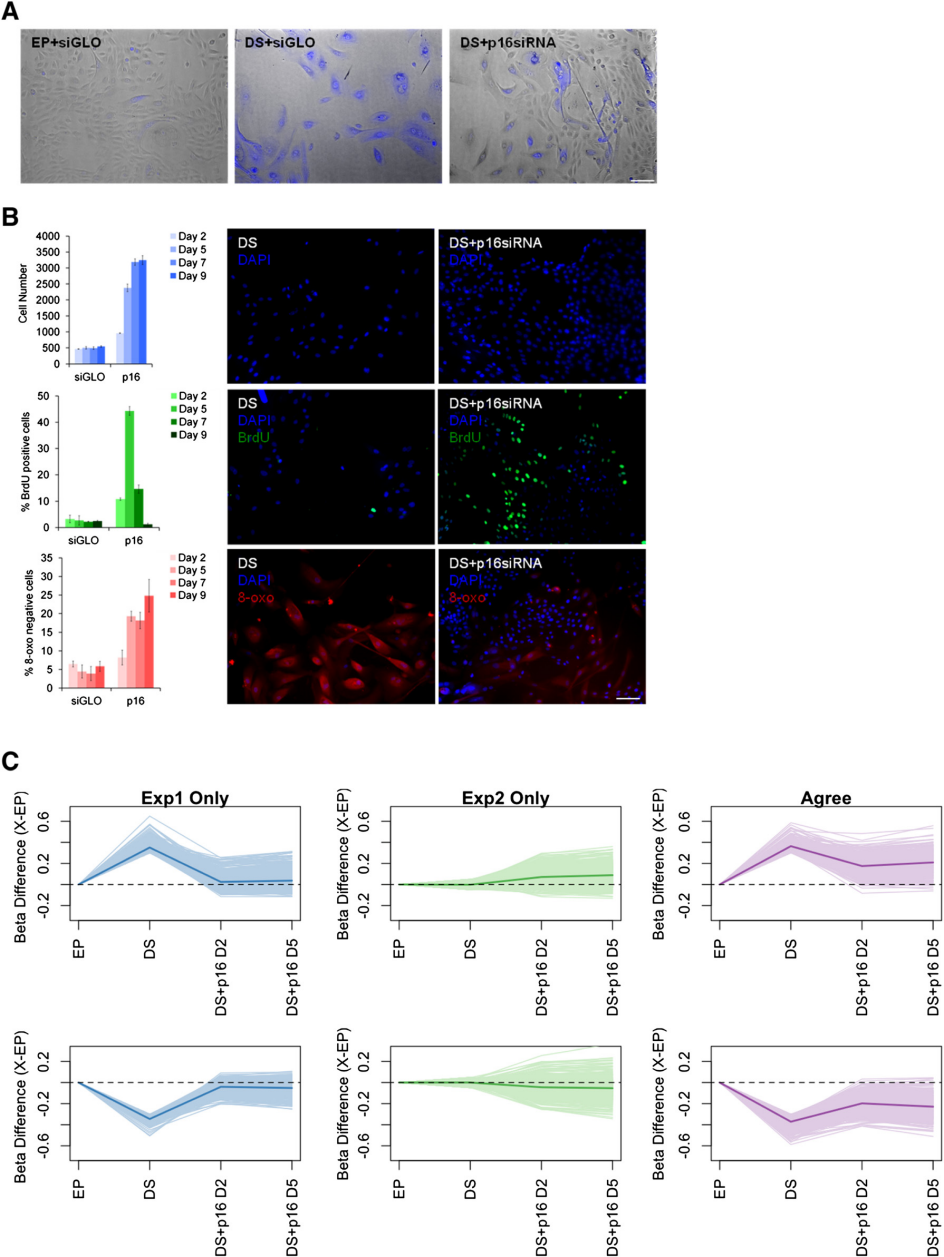
DNA methylation is reversed during the reversal of senescence of DS HMECs. **a** SA-β-gal staining of EP, DS+siGLO and DS+p16siRNA cells. **b** Quantitation of proliferation (top row), BrdU positivity (middle row), 8-oxoguanine negativity (bottom row) in DS+siGLO and DS+p16siRNA cells at days 2, 5, 7 and 9 post transfection. **c** A line plot of the beta value difference between each stage of the experiment and the EP cells for each of the hyper-senDMPs (top row) and hypo-senDMPs (bottom row). The thick coloured line in each plot represents the mean of all the hyper-senDMPs or hypo-senDMPs

The bypass of senescence typically describes a pro-tumourigenic escape from senescence (Additional file 1: Figure S1). The early proliferating cells that initially give rise to the senescent state are distinct from those that have overcome senescence [9, 14, 15]. By contrast, the senescent phenotype of adult human epithelial cells has long been considered irreversible, and a reversal of senescence to an EP state has not previously been demonstrated (Discussion). To examine this issue, we developed a highly efficient and reproducible protocol for introducing siRNA into DS cells (Additional file 1: Figure S2, Methods). Thereafter, we ablated p16 mRNA with a potent siRNA (Additional file 1: Figure S8, [16]), and assessed the impact on numerous cellular and molecular markers classically associated with senescence. A substantial fraction of the ‘DS+p16siRNA’ cells reverted to a morphology similar to EP cells, stained negative for SA-β-gal (Fig. 2a), and increased in cell number by six-fold over the 9-day timecourse. 5-bromo-2′-deoxyuridine (BrdU) incorporation, illustrative of transition through the S phase, was stimulated from 2.7 % ± 0.45 in DS cells to 10.8 % ± 0.44 and 44.3 % ± 1.68, at 2 and 5 days, respectively. By contrast, transfection with siGLO siRNA did not increase cell number or enhance the BrdU labelling-index over the time-course of the experiment (Fig. 2b). We also assessed a proxy marker for reactive oxygen species, 8-oxoguanine, and observed decreased 8-oxoguanine in the ‘DS+p16siRNA’ population (24.8 % ± 4.4, day 9), in contrast with DS cells, the majority of which remained positive. ‘DS+p16siRNA’ cells also displayed downregulation of the senescence-associated secretory phenotype (SASP) pro-inflammatory signature, as measured by decreased levels of the cytokines IL-6 and IL-8 (Additional file 1: Figure S9). Finally, we observed increased expression of members of the polycomb group of proteins (CBX7, EED, EZH2 and Suz12) which are typically downregulated during senescence (Additional file 1: Figure S10). Therefore, by every marker tested, cellular senescence appears to be largely reversed in ‘DS+p16siRNA’ cells. Although the phenotype is transient, these experiments appear to provide the first example of the reversal of senescence of adult human epithelial cells.

### Reversal of senescence-associated methylation dynamics

To examine global methylation dynamics of the senDMPs following senescence reversal, we established genome-scale DNA methylation profiles for three independent DS cultures derived from experiment 1 EP cells at 2 and 5 days post p16 siRNA transfection. Comparison of the methylation state of DS+p16siRNA (day 2) with the EP signature revealed that 90 % (656) of the agree sites a methylation state below the 0.3 threshold and 18 % (127) of sites are below 0.1. This suggests that the vast majority of agree sites have undergone some degree of reversal, but at the day 2 timepoint only 18 % exhibit complete reversal to the EP state. One hundred percent (969) of experiment 1 only sites have a methylation state below the 0.3 threshold and 86 % (834) are below 0.1 suggesting a more complete reversal of these sites (Fig. 2c). At day 5, DS+p16siRNA cells began to re-express p16 (mRNA, Additional file 1: Figure S8), and the methylation signature began to revert to the former DS pattern (Fig. 2d). Now only 76 % (553) of the agree sites are below 0.3 and 14 % (102) are below 0.1. For experiment 1 only sites 99 % (964) methylation state is still below 0.3 but now 79 % (768) are below 0.1.

The DS+p16 450 K samples were generated by transfecting three independent cultures passaged to DS from experiment 1 with p16 siRNA. Therefore, by definition, all of the 1,807 experiment 2 only sites were below the 0.1 threshold in the DS samples prior to senescence reversal (Fig. 1c). Interestingly, a number of these experiment 2 only sites displayed methylation dynamics in the DS+p16siRNA (day 2) samples, with 25 % (459) of sites above the 0.1 threshold, and show similar directionality to that found in experiment 2 between EP to DS (Kendall’s Coeffecient = 0.45, *P* value <2.2×10^−16^). At day 5, experiment 2 only sites also continue to move towards a DS pattern with 34 % (628) above the 0.1 threshold. During tumourigenesis, overcoming senescence typically occurs following hypermethylation of the p16 promoter [8], and this is commonly observed within the cancerous breast epithelium [17]. However, and importantly, during the reversal of senescence we observe no change in the methylation levels of CpG sites at the p16 promoter in EP, DS or DS+p16siRNA cells (beta value difference >0.3, data not shown). Overall, these data show that the reversal of senescence protocol effectively reversed the senDMP signature, and that senDMPs can indeed be considered as robust markers for the senescent state *per se*.

### SenDMPs show significant enrichment with expression changes in DS cells

**Fig. 3:**
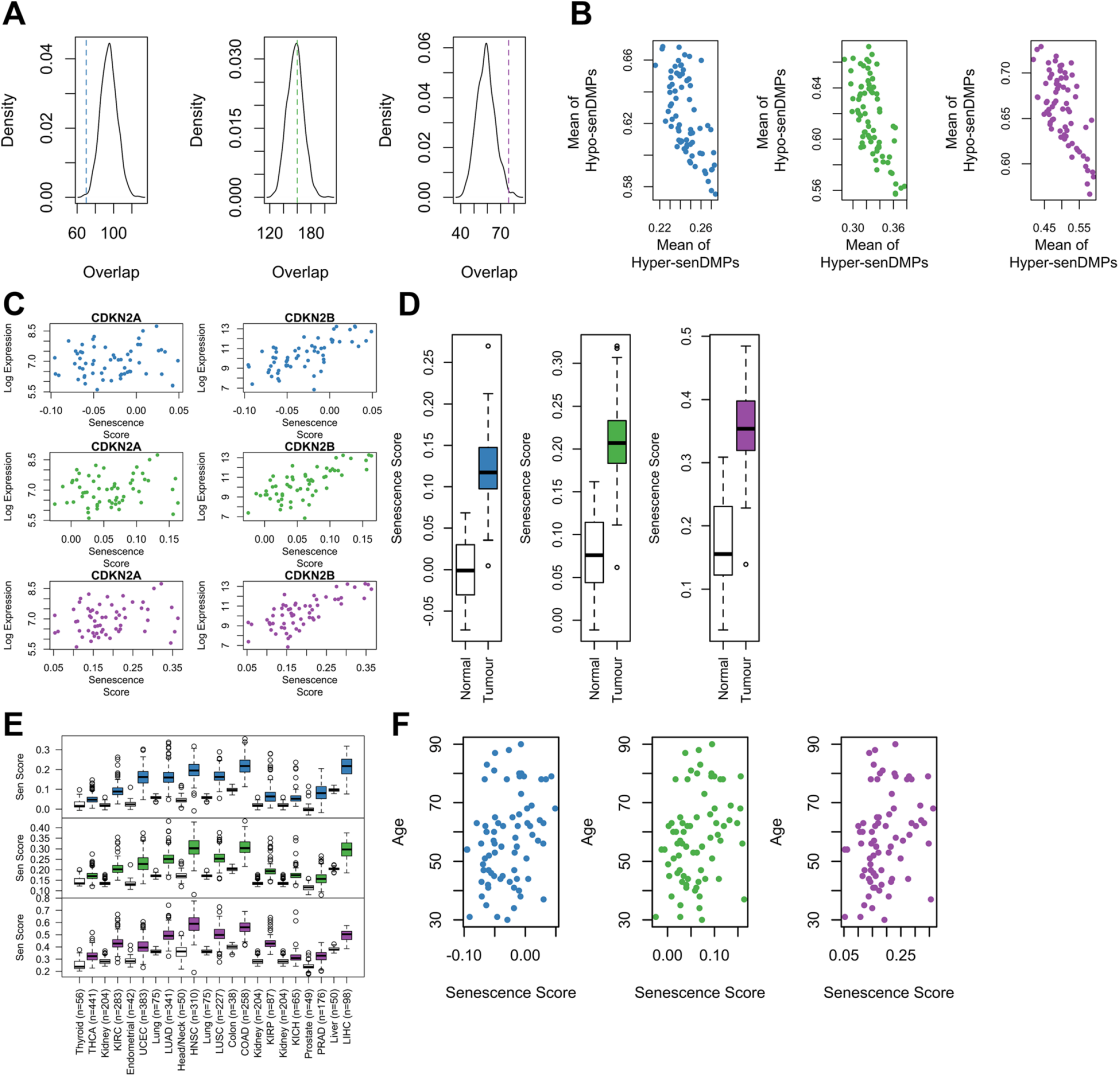
The methylomic signature of cellular senescence shows no association with expression but is shared among methylation signatures of human cancers and ageing. **a** The number of genes that are both significantly altered in both expression and methylation for senDMPs (coloured vertical line; blue – experiment 1 only, green – experiment 2 only, and purple – agree) against the background model. There is a significant enrichment of genes altered in both gene expression and methylation for those sites that agree while no significant enrichment for those sites which vary in experiment 1 or 2 only. **b** Scatter plot of the average methylation in normal breast tissue from TCGA for CpGs that are hypermethylated during senescence against those that are hypomethylated in senescence. The three different sites are plotted in three different panels going from left to right: blue – experiment 1 only, green – experiment 2 only, and purple – agree. A negative correlation implies that our senDMP signature is present in these breast samples. **c** The senescence score was calculated for each of the 73 normal breast tissues and plotted against the log of the expression for CDKN2A (left) and CDKN2B (right). The three different sites are plotted from top to bottom: blue – experiment 1 only, green – experiment 2 only, and purple – agree. **d** The senescence score was derived from senDMPs and calculated for both normal and tumour breast tissue from TCGA (n = 73). Tumour samples show a much higher senescence score compared to normal samples for the three different set of sites (blue – experiment 1 only, green – experiment 2 only, and purple – agree). **e** A boxplot of the senescence score for the samples from 11 different cancers (coloured boxes) calculated from the TCGA datasets and the corresponding control tissue for each cancer (white boxes). Each cancer sample shows an increased senescence score compared to the control samples. The three different sites are plotted from top to bottom: blue – experiment 1 only, green – experiment 2 only, and purple – agree. The 11 different cancers are Thyroid vThyroid Carcinoma (THCA), Kidney v Kidney renal clear cell Carcinoma (KIRC), Endometrial v Uterine Corpus Endrometrioid Carcinoma (UCEC), Lung v Lung Adenocarcinoma (LUAD), Head/Neck v Head/Neck Squamous Cell Carcinoma (HNSC), Lung v Lung Squamous Cell Carcinoma (LUSC), Colon v Colon Adenocarcinoma (COAD), Kidney v Kidney Renal Papillary Cell Carcinoma (KIRP) or Kidney Chromophobe (KICH), Prostate v Prostate Adenocarcinoma (PRAD), Liver v Liver Hepatocellular Carcinoma (LIHC). **f** Scatter plot of the senescence score for normal breast tissue against the age of the sample. A significant correlation between the senescence score and age is observed for all sets of sites, with older samples producing a higher senescence score. The three different sites are plotted from left to right: blue – experiment 1 only, green – experiment 2 only, and purple – agree

We investigated whether senDMPs showed any correlation with expression differences between EP and DS cells. We performed gene expression arrays in triplicate (IlluminaHT12) for EP and DS cells and asked whether promoter or gene-body DMPs are more likely to be associated with genes differentially expressed between EP and DS cells, regardless of the directionality. We found an enrichment of differentially expressed genes with those that were differentially methylated for sites which agreed between the two experiments (1.28-fold enrichment, *P* value = 0.012), while no such enrichment was observed for sites present in experiment 1 or 2 only (0.74- and 1.02-fold enrichment; *P* value = 0.999 and 0.413, Fig. 3a). We performed pathway analysis (Panther DB) for the agree sites and, although not all changes in expression were attributed to a particular pathway, this analysis revealed members of a total of 61 pathways, including a number of pathways considered to be hallmarks of senescence (Additional file 6: Table S7). These include the Inflammation mediated by chemokine and cytokine signaling pathway (P00031), and the Wnt Signalling pathway (P00057). Although the enrichment did not reach significance for experiment 1 and 2 sites, we were interested to determine the pathways with which these sites were associated (Methods, Additional file 7: Table S5 and Additional file 8: Table S6). Interestingly, this analysis highlighted a total of 37 common pathways between all three senDMP signatures, including P00031 and P00057 (37/61 for experiment 1, 37/81 for experiment 2 and 37/60 for agree sites). Finally, we asked if these 37 pathways had a function role in senescence by cross-referencing these with the 35 pathways that emerged from a previous genome-wide siRNA screen for novel modulators of senescence [16]. This revealed 17 pathways common to all three senDMP signatures as well as those that emerged from the siRNA screening, with further signature specific overlap (25/35 for experiment 1, 29/35 for experiment 2 and 25/35 for agree sites). Once again both P00031 and P00057 are present among these 17 pathways.

### Investigating the senDMP signature *in vivo*

We then investigated the potential *in vivo* relevance of the senDMP signature. As we derived the senDMP signature in HMECs, we used Illumina 450k data from 73 breast tissue samples labelled as normal in ‘The Cancer Genome Atlas’ (TCGA) project (Methods). For each normal breast sample, we calculated the average methylation observed at CpG sites identified as hypermethylated senDMPs (hyper-senDMPs) in HMECs, and separately the CpG sites identified as hypomethylated senDMPs (hypo-senDMPs). *A priori*, if there is an underlying biological process that links these CpG sites *in vivo* then methylation states at hyper-senDMPs should be negatively correlated relative to methylation states at hypo-senDMPs. For all groups of senDMPs we found a strong negative correlation (Kendall’s Coeffecient = −0.44 (experiment 1 only), −0.42 (experiment 2 only), −0.41 (agree); *P* value = 4.334×10^−8^, 1.988×10^−7^, 3.654×10^−7^, Fig. 3b) meaning that individuals who displayed higher methylation levels at hyper-senDMPs also displayed lower methylation levels at hypo-senDMPs. Using a similar number but randomly chosen probes, however, yields a strong positive correlation coefficient of 0.61 (Methods, Additional file 1: Figure S11).

Based on this observation, we defined a ‘senDMP score’ that conveys the strength of the correlation between the overall methylation pattern observed at senDMP-associated CpG sites, in an *in vivo* setting, with the senDMP signature we defined in HMECs (Methods).

We next wanted to establish if the *in vivo* senDMP scores in these 73 individuals were correlated with independent and well-established markers of senescence, namely expression levels of CDKN2A (p14 and p16) and CDKN2B (p15) [1]. We observed a non-significant (*P* value >0.05) positive correlation for CDKN2A within each of the three senDMP groups, although this gene showed considerably low levels of expression in all individuals. However, we found a striking positive correlation between our senescence score and the expression of CDKN2B in the normal breast tissue (Kendall’s correlation coefficient = 0.49 (experiment 1 only), 0.50 (experiment 2 only), 0.49 (agree); *P* value = 4.75×10^−8^, 2.82×10^−8^, 5.11×10^−8^, Fig. 3c). Importantly, in the original *in vitro* HMEC experiments, p15 expression was elevated in DS cells, and knockdown of p16 in DS cells resulted in reduced expression of p15.

We then investigated the senDMP signature in other tissues and found varying levels of negative correlation between hyper- and hypo-senDMPs (Additional file 1: Figures S13–S15). We acknowledged that this sendDMP signature was derived from a single tissue from a single individual and, as such, has inherit limitations. Thyroid produced the strongest negative correlation across all groups (Kendall’s correlation coefficient = −0.34 (experiment 1 only), −0.28 (experiment 2 only), −0.49 (agree); *P* value = 2.1×10^−4^, 2.7×10^−3^, 1.2×10^−7^) and hence seems to contain the strongest signal of senescence based on our senDMPs, while blood and prostate showed a positive correlation in line with the random sampling across all groups (Kendall’s correlation coefficient = 0.2 (experiment 1 only), 0.44 (experiment 2 only), 0.24 (agree); and 0.24 (experiment 1 only), 0.27 (experiment 2 only), 0.03 (agree), respectively), suggesting no association with our senDMP signature.

### The senDMP signature is associated with cancer

We then used the senDMP signatures to explore potential biological links with cancer. We calculated the senescence score for each of the three senDMP signatures in both tumour and normal breast samples from TCGA and found that tumours showed a marked increased measure of senescence (t-test = −20.6 (experiment 1 only), −20.0 (experiment 2 only), −20.0 (agree); *P* value <2.2×10^−16^ in all three, Fig. 3d). We then considered 11 further, very highly disparate cancers in TCGA for which there exists sufficient Illumina 450K data (>40 different matched samples per cancer, Fig. 3e). In all cases, tumour samples showed a significantly (t-test *P* value <4×10^−6^) greater senDMP score relative to matched healthy tissues.

### The senDMP signature is associated with *in vivo* ageing

Given recent work demonstrating that methylation is a strong marker for ageing [7], we wondered if our measure of senescence showed correlation with ageing. We therefore correlated our senescence score for each individual from the 73 breast tissue samples with their annotated age of sampling. We find that our senescence score shows a significant correlation with age (Kendall’s correlation coefficient = 0.21 (experiment 1 only), 0.20 (experiment 2 only), 0.22 (agree); *P* value = 0.011, 0.015, 0.0062, Fig. 3f), implying that older people have increased methylation changes at senDMPs. While these correlations are statistically significant, they are weaker than the correlation between age and CpGs directly associated with age [7].

### Common disease linked SNPs associated with the p16 locus are linked with senDMPs

**Fig. 4:**
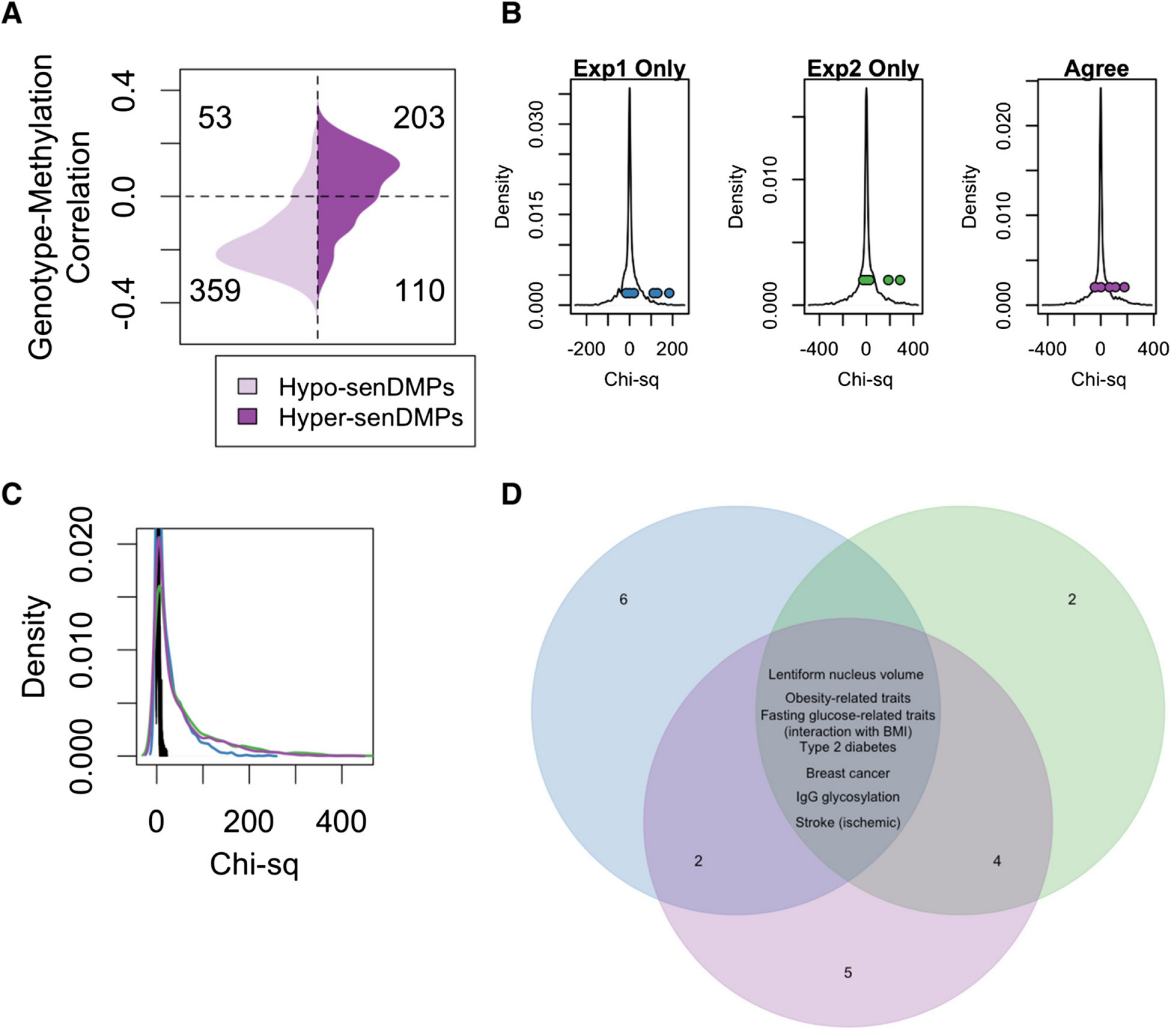
Common disease linked SNPs are correlated with senDMP signature. **a** For each of the senDMPs Pearson’s correlation coefficient was calculated for each SNP (in this figure rs10811661). We then compared the directionality of senDMPs (DS-EP – in this example the agree sites have been used) to the correlation with genotype (risk allele–alternative allele) and calculated a chi-squared. **b** A density plot of the chi-squared statistic for all (2,862) GWAS SNPs present on the genotype array. Highlighted in colour are those SNPs associated with the p16 locus of which two show significant directionality with our senDMPs across the three groups of senDMPs. These are plotted left to right: blue – experiment 1 only, green – experiment 2 only, and purple – agree. **c** A density plot of the chi-squared statistic for all GWAS SNPs measuring the association with senDMP signature (blue – experiment 1 only, green – experiment 2 only, and purple – agree) and 100 random shuffled signatures (black). **d** Venn diagram illustrating the traits associated with the SNPs which correlated with the senDMP signatures (blue – experiment 1 only, green – experiment 2 only, and purple – agree)

Based on the importance of p16 in the reversal protocol, we hypothesised that individuals with SNPs associated with the p16 locus (INK/ARF locus) should have measurably different DNA methylation profiles at senDMPs. To investigate this, we extracted methylation (Illumina 450K) and genotype data (Affymetrix Genome-Wide Human SNP Array 6.0) from TCGA. As our senDMP signature was originally found in HMECs, we focused on 73 normal samples from individuals with breast invasive carcinoma (Methods), and those SNPs (2,878) that have been reported in the GWAS catalogue [18]. For each of these we extracted the genotype for each of the 73 individuals and calculated the Pearson’s correlation coefficient of genotype with methylation for each of our 725 agree senDMPs (Fig. 4a). We then calculated a directional chi-squared statistic using only those senDMPs which showed a >0.15 methylation and genotype correlation (Methods). A minimum threshold of >0.15 was chosen to reduce potential spurious correlations (Additional file 1: Figure S16) although the results remain relatively unchanged without this cutoff (data not shown). We found two of the GWAS SNPs associated with the p16 locus to have significant (*P* value <0.1) association of genotype with our senDMP signature (Permutation *P* value: rs10811661 = 0.0014 (experiment 1 only), 0.014 (experiment 2 only), 0.033 (agree); rs2383208 = 0.010 (experiment 1 only), 0.043 (experiment 2 only), 0.081 (agree); rs1333051 = 0.013 (experiment 1 only), 0.040 (experiment 2 only), 0.13 (agree), Fig. 4b). These are associated with susceptibility to type 2 diabetes. As these all had similar chi-squared values and associated traits we investigated their linkage disequilibrium using the online database GLIDER [19] and found that these three SNPs were all in tight linkage.

Interestingly, other GWAS SNPs showed an even stronger correlation with the senDMP signature compared with p16-associated SNPs, suggesting the presence of multiple genetic variants that can influence the senDMP signature *in vivo* (Table 1). To confirm this, we permutated the labels of the senDMPs and re-calculated the correlations between GWAS SNPs and these permuted senDMPs (1,000 different permutations). No SNPs in any of the permutations showed high levels of correlation (>100 Chi-squared statistic), whereas the true labels resulted in 471 GWAS SNPs associated with an *in vivo* senDMP signature (Fig. 4c). Seven common traits emerged for the three senDMP sigantures: Type 2 diabetes, ischaemic stroke, breast cancer, lentiform nucleus volume, fasting glucose-related traits (interaction with BMI), obesity-related traits and IgG glycosylation (Fig. 4d).

**Table 1:** Top 10 GWAS SNPs which are associated with senDMP signature ordered by absolute chi-squared statistic

SNP ID	CHR	Position	Gene	RAL	Trait	Cor	Stat
rs10496584	2	123126479	Intergenic	A	Lentiform nucleus volume	0.885	−424
rs4848768	2	123141545	Intergenic	B	Lentiform nucleus volume	−0.865	−403
rs12716852	16	78188738	WWOX	A	Pulmonary function	0.896	−375
rs17041	6	108853493	LACE1	B	Obesity-related traits	0.927	369
rs7944584	11	47336320	MADD	A	Fasting glucose-related traits (interaction with BMI)	0.909	−354
rs2989476	1	61059259	NFIA	A	Bipolar disorder	−0.888	338
rs966423	2	218310340	DIRC3	A	Thyroid cancer	0.869	−338
rs4527850	8	134196849	WISP1	A	Type 2 diabetes	0.839	−327
rs4432842	5	57172078	Intergenic	B	Birth weight	0.810	326
rs2479106	9	126525212	NR	B	Polycystic ovary syndrome	0.860	326

Cor: Pearson’s correlation coefficient between the SNP and the senDMPs; RAL: risk allele for the SNP; Stat: the directional chi-squared statistic

## Discussion

Here we have identified CpG sites (senDMPs) that displays significant methylation dynamics between actively dividing and deeply senescent human mammary epithelial cells. The senDMP signatures correlates with cancer and ageing methylomes, are influenced by genetic variation, and displays inter-individual variation in unaffected tissues. Crucially, the senDMP signatures can be essentially reversed upon reversal of senescence.

The observation that non-proliferative, deeply senescent cells share a partially common signature of DNA methylation with diverse cancers is unexpected. Advanced tumours are highly heterogeneous and include not only genetically-damaged tumour cells but also induced vasculature, infiltrating fibroblasts and inflammatory cells. High levels of p16 expression, and thus possibly senescent cells, have been widely reported in such non-tumour cells [20, 21]. However, the strength of the cancer and senDMP relationship more likely reflects the properties of the tumour tissue itself. It has been suggested that senescent cells create an environment conducive to tumourigenesis [22], and indeed that tumours may arise directly from cells that had reached senescence and then overcome this programme following a pro-tumourigenic event. If so, the senescent cell DNA methylation signature might simply carry over into the progressing tumour, and be maintained through the acquisition of mutations that generate common tumour hallmarks, such as p16 gene mutations. In support of this, recent work has shown that senescent fibroblasts also have methylation changes similar to those observed in cancer [23]. Importantly, the authors show that these changes are largely retained when cells overcome senescence following expression of SV40 T antigen. An alternative overall interpretation is that the methylation pattern may reflect underlying similarities in the molecular physiology of senescent and tumour cells, that implies no direct lineage relationship, but rather that each has reached, or exceeded, its natural ‘division potential’.

As recently demonstrated by several groups, DNA methylation now represents the single best molecular predictor of human age. Hannum *et al*. defined 71 different CpG sites that can predict chronological age to ±4 years and 96 % accuracy [7]. We show a significant and positive correlation between the senDMPs signature and age of the sample, but also imply that senescence scores are not simply related to a person’s age. This idea is supported by our observation that human individuals display varying degrees of correlation with the senDMP signature, which in turn correlates well with known *in vivo* markers of senescence such as CDKN2A/2B expression levels. Does this inter-individual variation in senescence scores imply population-level variation in the proportion of senescent cells present in different individuals? If so, what are the relative contributions of ageing, environment and genetics? Our analysis shows, for the first time, that genetic variation in the human population could potentially influence the degree of *in vivo* senescence. However, this needs to be explored in more detail in larger sample sets as they become available. Although p16-associated SNPs showed significant correlation with *in vivo* senDMP signatures, there were other additional SNPs, located throughout the genome, whose genotype showed an even greater correlation. Among these are a further type 2 diabetes SNP on Chr8 [24] and two SNPs on Chr2 associated with lentiform nucleus volume [25], deficits of which having previously been associated with normal ageing [26]. There is growing evidence that DNA methylation plays a role in disease susceptibility, which includes correlations between DNA methylation and common diseases [27, 28], and the genotype-dependent effect of allele-specific DNA methylation [29]. It is tempting to speculate that genetically driven cellular senescence may be a key factor in the etiopathogenesis of various diseases such as diabetes and cancer.

We observed an overall correlation between senescence-associated DNA methylation and gene expression dynamics for the agree sites. Pathway analysis (Panther DB) revealed a total of 17 pathways which were common to all three senDMP signature sets and those that emerged from a recent genome-wide siRNA screen for modulation of senescence. These included the Inflammation mediated by chemokine and cytokine signaling pathway (P00031) and the Wnt Signalling pathway (P00057). Inflammatory mediators are intimately linked with senescence and the senescence-associated secretory phenotype [22, 30] and this finding is in line with a recent work examining the senescent methylome of human fibroblasts [23]. Likewise Wnt signalling has an important role in restraining senescence in epithelial [16] pulmonary [31] and mammary stem cells [32].

In this work, we defined three sets of senDMPs referred to as experiment 1, experiment 2 and agree sites. Experiments 1 and 2 were each performed in triplicate using the same original cell stocks, however, they were performed 1 year apart using different batches of medium/supplements. One interpretation of the differences between experiments 1 and 2 is simply inter-experimental variation, and that only those sites which change in both set experiments (the agree sites) are important for the process. However, both experiments 1 and 2 senDMP signatures have a significant correlation with ageing and show the same negative correlation *in vivo* to that of agree sites. Furthermore, we find that those sites found in experiment 2 which do not change between DS and EP state in experiment 1, do change in the expected direction (hyper and hypo) at 2 days and 5 days after p16 knockdown. This may suggest that there are multiple ways in which a cell can senescence, and that deviation from a common starting point may be reflected at the level of the methylome. Interestingly we do find that those agree sites are not reversed to the same extent as the other senDMPs suggesting that these could be part of this initial common starting point. The finding that tumours from 12 different tissues show a greater senDMP score relative to healthy tissue for all three senDMP signatures may also indicate potential inter-individual variation which is captured by the full complement of senDMPs.

**Fig. 5:**
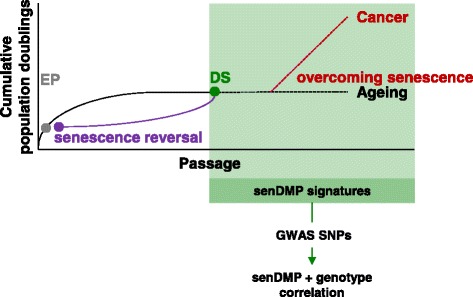
Model of reversal of senescence. Following serial passage, normal HMEC cultures eventually undergo p16-mediated cellular senescence and enter a state of deep senescence (DS). In this work, we have identified senDMP signatures and demonstrate that these are specific to the DS state and not the p16 status of the cells *per se*. Human senDMP signatures shows strong positive correlations with cancers from a broad range of tissues as well as ageing-associated methylomic dynamics. Senescence acts as an essential barrier to tumour progression. Overcoming this cellular programme, in combination with additional mutations, is believed to give rise to cancer. One possible interpretation of these findings is that cells which overcome senescence retain this senDMP signature as they progress towards cancer. We also describe the reversal of senescence, during which the senDMP signature essentially reverts to that seen in early proliferating (EP) cultures. This demonstrates that the senDMP signature has the potential to be dynamic

Finally, we also show that the senescent phenotype should not be considered irreversible. The reversibility of p16-mediated cellular senescence, and the associated pattern of DNA methylation, is unequivocal. The distinction between reversal and overcoming senescence is critical, the latter being associated with a hypermethylation of the p16 promoter [8]. Upon reversal, the senDMP signature essentially reverts to that seen in EP cells. It is important to note that this occurs in the absence of any significant changes in the methylation status of the p16 promoter. Reversal of senescence has been described in embryonic murine fibroblasts [33, 34], and in human fibroblasts of fetal origin [35, 36] that are arrested by p53 rather than p16. However, rescue of adult human cells from senescence has not been described previously. Given that transgenic mice in which p16 is specifically inactivated in particular tissues (for example, pancreatic β-cells [37] and haemopoetic stem cells [38]) display improved regenerative capacity in aged animals, the tissue culture model of reversal of senescence that we describe here may be used as a starting point for elaboration of pathways of cellular rejuvenation (see Fig. 5, model) and as more 450 K data from different tissues becomes available to further explore and refine the pan-tissue signatures.

## Conclusions

This work identified senDMPs that display significant methylation dynamics during senescence and our newly developed senescence reversal protocol. This senescent methylome correlated with *in vivo* cancer and ageing, and with the expression of senescence markers. Finally, we provide evidence indicating that the *in vivo* senescent methylome may be influenced by germline genetic variation. In summary, we propose that senDMPs represent a highly relevant signature that will enable novel *in vivo* investigations into the role of cellular senescence in human ageing-related phenotypes and diseases, including cancers.

## Materials and methods

### Cells and reagents

Normal finite life span HMECs were obtained from reduction mammoplasty tissue of a 21-year-old individual, specimen 184, and were cultured as previously described (Garbe *et al.* [9]). The HMEC specimen (Specimen 184) was obtained in 1980. This was before the current IRB regulations were in place, and consent at that time was covered by the hospitals’ consent forms which allowed the pathologists to use or distribute discard surgical material (which is what we obtained) at their discretion. Independent cultures from this individual were serially passaged from passage 6 (early proliferating, EP cells) through to deep senescence (DS cells). DS cultures underwent no further expansion upon at least two serial passages.

### siRNA transfections

A panel of scrambled siRNAs were tested as negative controls in the HMECs, however, each of these induced some degree of toxicity or phenotypic change in the EP cultures. The fluorescently labelled siRNA targeting cyclophilin B (siGLO) was selected for the experiments presented as this did not influence the phenotype of either EP or DS cultures. HMECs were transfected with 60 nM siGLO siRNA (Dharmacon) or p16 siRNA (Qiagen) in 384-well or 6-well plates using Dharmafect3 (Dharmacon). EP+siGLO, DS+siGLO or DS+p16siRNA cells were harvested for flow cytometry, DNA extraction or immunofluorescence as detailed below.

### Flow cytometry

EP+siGLO cells were harvested by trypsinisation 48 h post transfection. Cells were fixed using 4 % paraformaldehyde + 5 mM EDTA, followed by permeabilisation with 0.1 % Triton-X 5 mM EDTA. Cells were blocked in 0.25 % BSA 5 mM EDTA 0.25 % saponin, and then incubated on ice with mouseαp16 JC2 (1:1,000), goatαmouse AlexaFlur-647 (1:500, Invitrogen) and finally DAPI. Cells were sorted into G1-p16^–ve^, G2/S-p16^–ve^ and G1-p16^+ve^ fractions based on DNA content and isoform-matched control antibodies conjugated to goatαmouse AlexaFlur-647 using the ARIA II (Becton Dickinson).

### Immunofluorescence

Standard fixation with 3.7 % paraformaldehyde, followed by 0.1 % Triton X permeabilisation and blocking with 0.25 % BSA was performed prior to antibody incubations. Primary antibodies used were mouseαp16 JC2 (1:1,000) or mouseα8-Oxoguanine (1:100, MAB3560 Millipore), followed by goatαmouse AlexaFlur-488 (1:500, Invitrogen), DAPI and Cell Mask Deep Red (1:10,000, Invitrogen). For 5-bromo-2′-deoxyuridine (BrdU) assays, cells were cultured in 0.4 μM BrdU for 16 h prior to fixation. An additional DNA denaturation step with 4 M HCl for 10 min was performed following permeabilisation, and a conjugated mouseαBrdU-AlexaFlur-488 antibody (1:100, B35130 Invitrogen) used. Images were collected at 10× using the IN Cell 1000 microscope (GE) and the Developer Analysis software (GE) was used for image analysis as described previously (Bishop *et al.* [16]).

### Processing of arrays

For Illumina 450K methylation arrays IDAT files were processed using the R package minfi [39]. Quantile normalisation was performed on the intensity values of the red and green channels of the type I and type II probes separately. Probes with a detection *P* value <0.01 or those mapping to more than one location (with 90 % similarity) or to the X or Y chromosome were removed from further analysis, leaving 401,915 probes (experiment 1) and 431,909 probes (experiment 2). The X chromosome was removed to allow for consistent analysis across a large number of datasets. Intensities were combined into a single beta value. We also performed the analysis and called senDMPs only removing the Y chromosome and those DMPs found to be significantly changed with >0.3 beta value difference and located on chrX are included in Additional file 9: Table S4. For IlluminaHT12 RNA arrays, the samples were processed using the lumi package. Arrays were first transformed using the Variance Stabilizing Transform and then normalised using robust spline normalisation. All further analysis was performed on the normalised transformed data.

### Identification of senDMPs

DMPs were called using the dmpFinder function available in the R package minfi using the categorical variable method. This function uses an F-Test to calculate a *P* value between two or more groups and then calculates a corrected *P* value (q-value). For all differential methylated positions we filtered first by q-value <0.01 and then by an absolute beta difference >0.3 unless otherwise stated in the manuscript.

### SNP analysis

We focused on those SNPs (2,878) that have been reported in the GWAS catalogue [18] downloaded on 2 December 2013. For each SNP we extracted the genotype from the birdseed files downloaded from TCGA for each of the 73 individuals. We then calculated for each SNP the Pearson’s correlation coefficient of the genotype of the 73 individuals with the methylation for each of our 3,852 senDMPs (for example, we calculated 3,852 correlations for each SNP). We then set a minimum threshold of >0.15 correlation (Additional file 1: Figure S12) and calculated a chi-squared statistic by assigning each senDMP to a bin of either hypermethylated or hypomethylated between DS and EP cells and either positively correlated or negatively correlation of the methylation with the risk allele of the SNP. Then those SNPs in which the risk allele showed a methylation signature similar to DS cells we assigned as a positive chi-squared and those that the risk allele showed a methylation signature similar to EP a negative chi-squared.

### Defining differences in DS across experiments

To investigate the differences of the DS cells across experiments, we characterised the differential probes into four categories: (1) Batch effect defined by an absolute beta value difference of less than 0.1 between EP and DS within an experiment (777); (2) Unique changes between DS and EP within an experiment defined as an absolute beta value difference between DS and EP in one experiment greater than 0.1 and in the other less than 0.1 (6,390); (3) Those changes between DS and EP in common between experiments defined as an absolute beta vale difference >0.1 in both experiments and in the same direction (314); and (4) Those changes between DS and EP in common between experiments but in opposite direction defined as beta value difference >0.1 in both experiments and in the opposite direction (4,087).

### TCGA data

Methylation and expression data were downloaded from the TCGA public repository [40] using custom scripts. For methylation arrays idat files were downloaded and samples were removed which showed low (<80 %) bisulfite conversion efficiencies. The remaining samples were then processed in the same manner as above. For expression data, processed data termed level 3 were used for all analysis. SNP array data were accessed using the private repository and birdseed files were downloaded for each of the individuals. All normal samples used in this analysis from TCGA data are matched normal samples, for example, the healthy tissue from the same individual.

### Genomic feature analysis

Average methylation was calculated as the average of the beta values for the triplicate EP cells for each of the probes. The CpG density was calculated by taking a 400 bp window around the probe and calculating the number of Cs, Gs and CpGs contained in that window using reference genome hg19. The fold enrichment or background models were always performed by randomly selecting the same number of probes as the DMPs in question and repeatedly re-sampling 1,000 times. Genomic location of the Illumina 450K probes was extracted using the annotation as provided by Illumina. For histone enrichment a number of histone marks for HMEC were downloaded from [41] in BED format and overlapped using BEDTools with DMPs.

### Panther DB analysis

The unique gene names associated with the experiment 1 only, experiment 2 only and agree senDMPs were used as the input gene list and the unique gene names associated with the 401,915 (experiment 1 only), 431,909 probes (experiment 2) and 391,341 (agree) probes analysed in this experiment were used as the background gene list. These lists were uploaded to Panther DB [42] and pathway analysis was performed (Additional file 7: Table S5, Additional file 8: Table S6 and Additional file 6: Table S7).

### Co-methylation analysis

To calculate the co-methylation of each of the DMPs we first calculated the nearest probe to each of the called DMPs. We then calculated the beta difference for the nearest probes for each original probe called either hypermethylated or hypomethylated. This means that a called probe may actually be the closest neighbour to another called probe and in this case will be used as the neighbouring probe. Removing such probes would cause a bias and underestimation of the co-methylation. To create a background model we randomly sampled the same number of probes as were originally called and repeated the exact same process for these probes. We repeated this a number of times, each time re-sampling the originally chosen probes.

### Defining an *in vivo* senescence score

We used our initial cell line experiment to derive 969 (experiment 1 only), 1,807 (experiment 2 only) or 725 (agree) distinct senescence-associated DMPs of which 679, 1,067 and 313 were hypermethylated (hyper-senDMPs), respectively, and 290, 740 and 412 were hypomethylated (hypo-senDMPs), respectively. To investigate this signature of these senDMPs *in vivo* we extracted the matched normal breast tissue samples of 73 individuals from ‘The Cancer Genome Atlas’ (TCGA) and investigated the relationship between the hyper-senDMPs and hypo-senDMPs in these samples. We hypothesised that if this signature was a measure of senescence then an individual sample with a higher level of senescence would show increased methylation at hyper-senDMPs, conversely they should also show decreased methylation at hypo-senDMPs compared to an individual with a lower level of senescence. It is highly likely that not all the senDMPs derived *in vitro* will be a strong measure of senescence *in vivo* and hence we used the mean beta value of hyper-senDMPs and hypo-senDMPs to reduce this potential noise. Additional file 1: Figure S12 shows that this mean beta value captures the shift in distributions of the beta values of both the hyper-senDMPs and hypo-senDMPs across our samples. We found a highly significant and strong negative correlation of the mean beta values of hyper and hypo-senDMPs and hence we defined a ‘senDMP score’ (S) by combing these two values for each individual using the equation below:

$$ S=\frac{1}{n_{hype r}}{\displaystyle \sum_{i=1}^{n_{hype r}}{B}_{hype{r}_i}}-\frac{1}{n_{hypo}}{\displaystyle \sum_{i=1}^{n_{hypo}}{B}_{hyp{o}_i}}+0.35 $$

Where ${B}_{hype{r}_{ik}}$ represents the beta value of the ith hyper-senDMP of the kth individual, *n*_*hyper*_ represents the number of hyper-senDMPs, ${B}_{hyp{o}_{ik}}$ represents the beta value of the ith hypo-senDMP of the kth individual and *n*_*hypo*_ represents the number of hypo-senDMPs. The value of 0.35 is added to produce a senescence score close to 0 for the lowest score of the 73 individuals. A higher score of senescence implies that an individual shows higher methylation at hyper-senDMPs and lower methylation at hypo-senDMPs.

### Data accessibility

The complete methylation and expression profiles are available at the Gene Expression Omnibus (GSE58035).

## Additional files

Additional file 1: Figure S1. Model of senescence barriers in cultured HMECs. **Figure S2.** Morphology of DS cells following transfection. **Figure S3.** Comparison of the methylation state between EP and DS technical replicates for experiment 1. **Figure S4.** Comparison of the methylation state between EP and DS technical replicates for experiment 2. **Figure S5.** Comparison of average methylation between experiment 1 and 2 for EP and DS cells. **Figure S6.** Genomic feature analysis of senDMPs. **Figure S7.** The FACs sorting protocol. **Figure S8.** Decreased expression of p16 following reversal. **Figure S9.** Decreased expression of IL-6 and IL-8 following reversal. **Figure S10.** Increased expression of Polycomb proteins following reversal. **Figure S11.** Random permutation tests for hyper and hypo methylated Cpg sites. **Figure S12.** Average beta values for senDMPs in normal breast tissue samples. **Figure S13.** Average beta values for experiment 1 only senDMPs in eight different tissues. **Figure S14.** Average beta values for experiment 2 only senDMPs in eight different tissues. **Figure S15.** Average beta values for agree only senDMPs in eight different tissues. **Figure S16.** Plot showing the fraction of hypersenDMPs that showed a positive methylation and genotype correlation and hypo-senDMPs that showed a negative methylation genotype correlation (red) for different correlation cut offs. (DOC 2.43 mb) Additional file 2: Table S1. Table containing the annotated information of the 969 experiment 1 only senDMPs. (CSV 290 kb) Additional file 3: Table S2. Table containing the annotated information of the 1,807 experiment 2 only senDMPs. (CSV 539 kb) Additional file 4: Table S3. Table containing the annotated information of the 725 agree senDMPs. (CSV 244 kb) Additional file 5: Table S8. Table containing the *P* values for the difference in methylation between the EP cells and each of the FACs sorted populations as well as the FACs sorted populations against each other. (CSV 89758 kb) Additional file 6: Table S7. Panther DB pathway analysis of the 725 agree senDMPs. (XLSX 11 kb) Additional file 7: Table S5. Panther DB pathway analysis of the 969 experiment 1 only senDMPs. (XLSX 11 kb) Additional file 8: Table S6. Panther DB pathway analysis of the 1,807 experiment 2 only senDMPs. (XLSX 12 kb) Additional file 9: Table S4. Table containing the annotated information of the senescence associated probes reaching 0.01 FDR and beta value difference >0.3 in both experiment 1 and experiment 2 which are located on chrX. (CSV 1 kb)
