# Author Correction: The senescent methylome and its relationship with cancer, ageing and germline genetic variation in humans

**DOI:** 10.1186/s13059-024-03295-7

**Published:** 2024-06-10

**Authors:** Robert Lowe, Marita G. Overhoff, Sreeram V. Ramagopalan, James C. Garbe, James Koh, Martha R. Stampfer, David H. Beach, Vardhman K. Rakyan, Cleo L. Bishop

**Affiliations:** 1 The Blizard Institute, Barts and The London School of Medicine and Dentistry, Queen Mary University of London, 4 Newark Street, London, E1 2AT UK; 2 Life Science Division, Lawrence Berkeley National Laboratory, Berkeley, CA 94720 USA; 3 Division of Surgical Sciences, Department of Surgery, Duke University Medical School, Durham, NC 27710 USA

**Author Correction: Genome Biol 16, 194 (2015)**

**https://doi.org/10.1186/s13059-015-0748-4**

Following publication of the original article [1], the authors reported two errors in Fig. 1B and Fig. 2B.

1. The two EP + siGLO and DS + siGLO brightfield/β-gal images from Fig. 2A were accidentally duplicated in Fig. 1B. The images in Fig. 2A are correct.

The images in Fig. 1B are replaced with representative images of untransfected EP and DS cells. The incorrect and the correct figure are given below.

The incorrect Fig. 1:
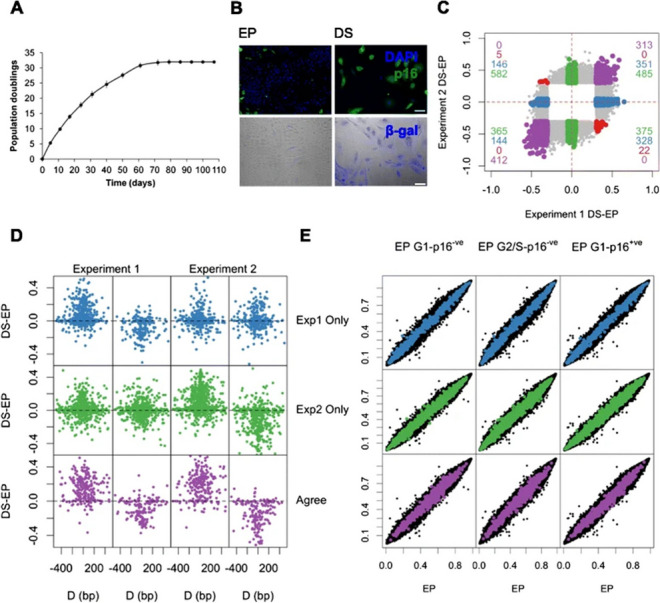


The correct Fig. 1:
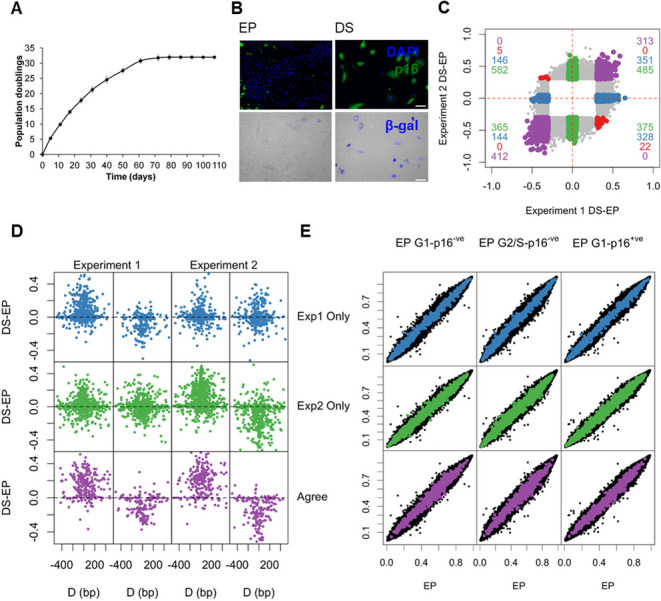


The replacement of these images does not change any of the other data in this figure, nor the conclusion of this figure.

2. The authors also noticed that the panels in Fig. 2B labelled with “DS” were incorrectly labelled. The bar charts and figure legend correctly state that these images are “DS + siGLO” images. These panels are now relabelled as “DS + siGLO”.

This proposed relabelling does not change any of the other data in this figure, nor the conclusion of Fig. 2B. The incorrect and the correct figure are given below.

The incorrect Fig. 2:
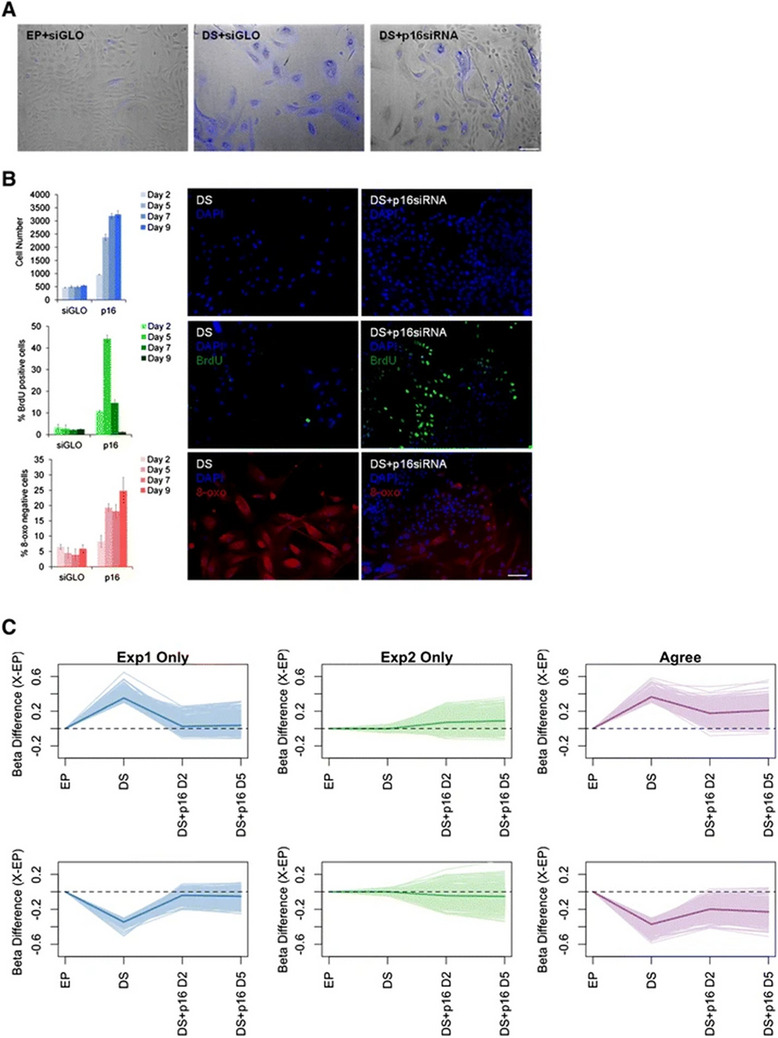


The correct Fig. 2:
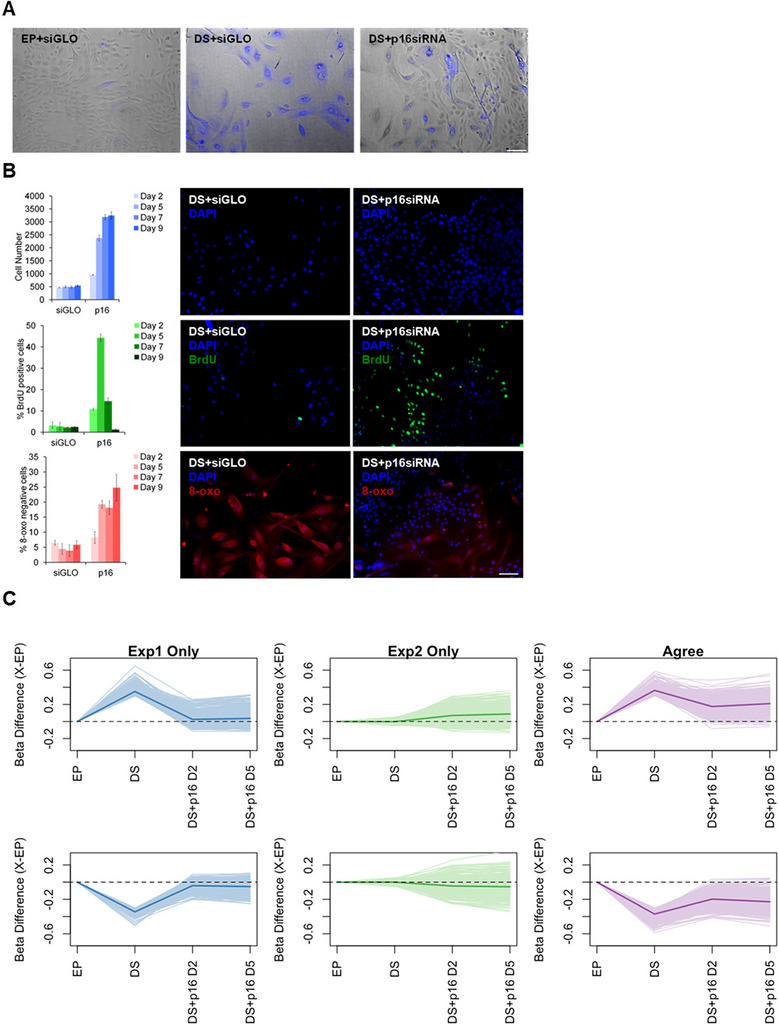


In both instances, these errors do not change any data associated with these figures, nor do they change the conclusions of the corresponding figures and the paper.
