# Supplementary material for: The senescent methylome and its relationship with cancer, ageing and germline genetic variation in humans
Source: Genome Biol. 2015 Sep 17;16(1):194. doi: 10.1186/s13059-015-0748-4 (PMC4574115; doi:10.1186/s13059-015-0748-4)
Supplement: Additional file 1: Figure S1. — Model of senescence barriers in cultured HMECs. Figure S2. Morphology of DS cells following transfection. Figure S3. Comparison of the methylation state between EP and DS technical replicates for experiment 1. Figure S4. Comparison of the methylation state between EP and DS technical replicates for experiment 2. Figure S5. Comparison of average methylation between experiment 1 and 2 for EP and DS cells. Figure S6. Genomic feature analysis of senDMPs. Figure S7. The FACs sorting protocol. Figure S8. Decreased expression of p16 following reversal. Figure S9. Decreased expression of IL-6 and IL-8 following reversal. Figure S10. Increased expression of Polycomb proteins following reversal. Figure S11. Random permutation tests for hyper and hypo methylated Cpg sites. Figure S12. Average beta values for senDMPs in normal breast tissue samples. Figure S13. Average beta values for experiment 1 only senDMPs in eight different tissues. Figure S14. Average beta values for experiment 2 only senDMPs in eight different tissues. Figure S15. Average beta values for agree only senDMPs in eight different tissues. Figure S16. Plot showing the fraction of hypersenDMPs that showed a positive methylation and genotype correlation and hypo-senDMPs that showed a negative methylation genotype correlation (red) for different correlation cut offs. (DOC 2.43 mb) [file 13059_2015_748_MOESM1_ESM.doc]

**Supplementary Information for:**

**The Senescent Methylome and its relationship with cancer, ageing, and germline genetic variation in humans**

Robert Lowe^1^, Marita G Overhoff^1^, Sreeram V Ramagopalan^1^, James C Garbe^2^, James Koh^3^, Martha R Stampfer^2^, David H Beach^1^, Vardhman K Rakyan^1^ and Cleo L Bishop^1^

^1^ The Blizard Institute, Barts and The London School of Medicine and Dentistry, Queen Mary University of London, 4 Newark Street, London E1 2AT, UK.

^2^ Life Science Division, Lawrence Berkeley National Laboratory, Berkeley, CA 94720, USA.

^3^ Division of Surgical Sciences, Department of Surgery, Duke University Medical School, Durham, NC 27710, USA.

^1^ The Blizard Institute, Barts and The London School of Medicine and Dentistry, Queen 
Mary University of London, 4 Newark Street, London E1 2AT, UK.

^2^ Life Science Division, Lawrence Berkeley National Laboratory, Berkeley, CA 94720, USA

Correspondence to: [v.rakyan@qmul.ac.uk](mailto:v.rakyan@qmul.ac.uk) or [c.l.bishop@qmul.ac.uk](mailto:c.l.bishop@qmul.ac.uk)


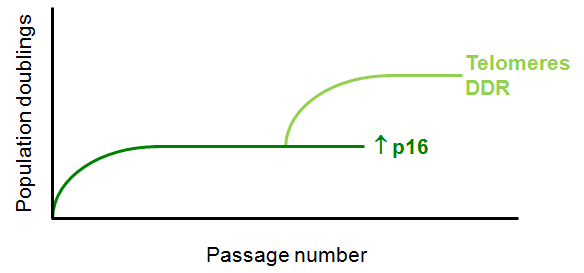


**Figure S1. Model of senescence barriers in cultured HMECs.** Following serial passage, normal HMEC cultures eventually undergo p16-mediated cellular senescence. These cells maintain a viable G1 cell cycle arrest, normal karyotype, low BrdU labeling index and are senescence-associated β-galactosidase positive. Cells that overcome of this barrier, for example by methylation of the p16 promoter or inactivation of RB, undergo a second phase of proliferation until they reach p53-mediated replicative senescence associated with critical short telomeres, DNA damage response (DDR), genomic instability and an abnormal karyotype.


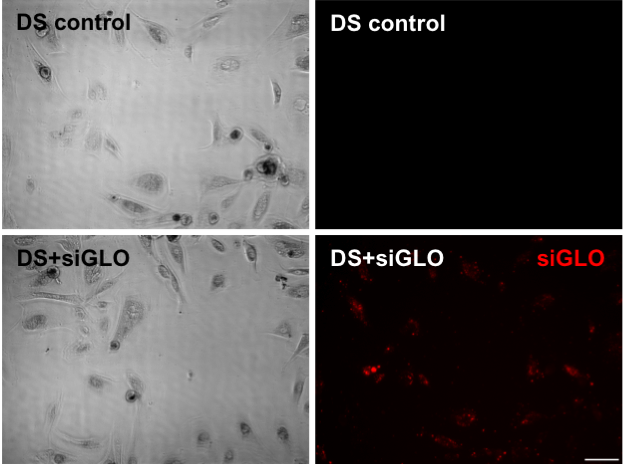


**Figure S2. Morphology of DS cells following transfection.** Cellular morphology of untransfected DS cells (DS control) versus siGLO transfected cells (DS+siGLO) (left panels). The right hand panels show excitation of the fluorescently labeled siGLO siRNA. Size bar, 100μm.


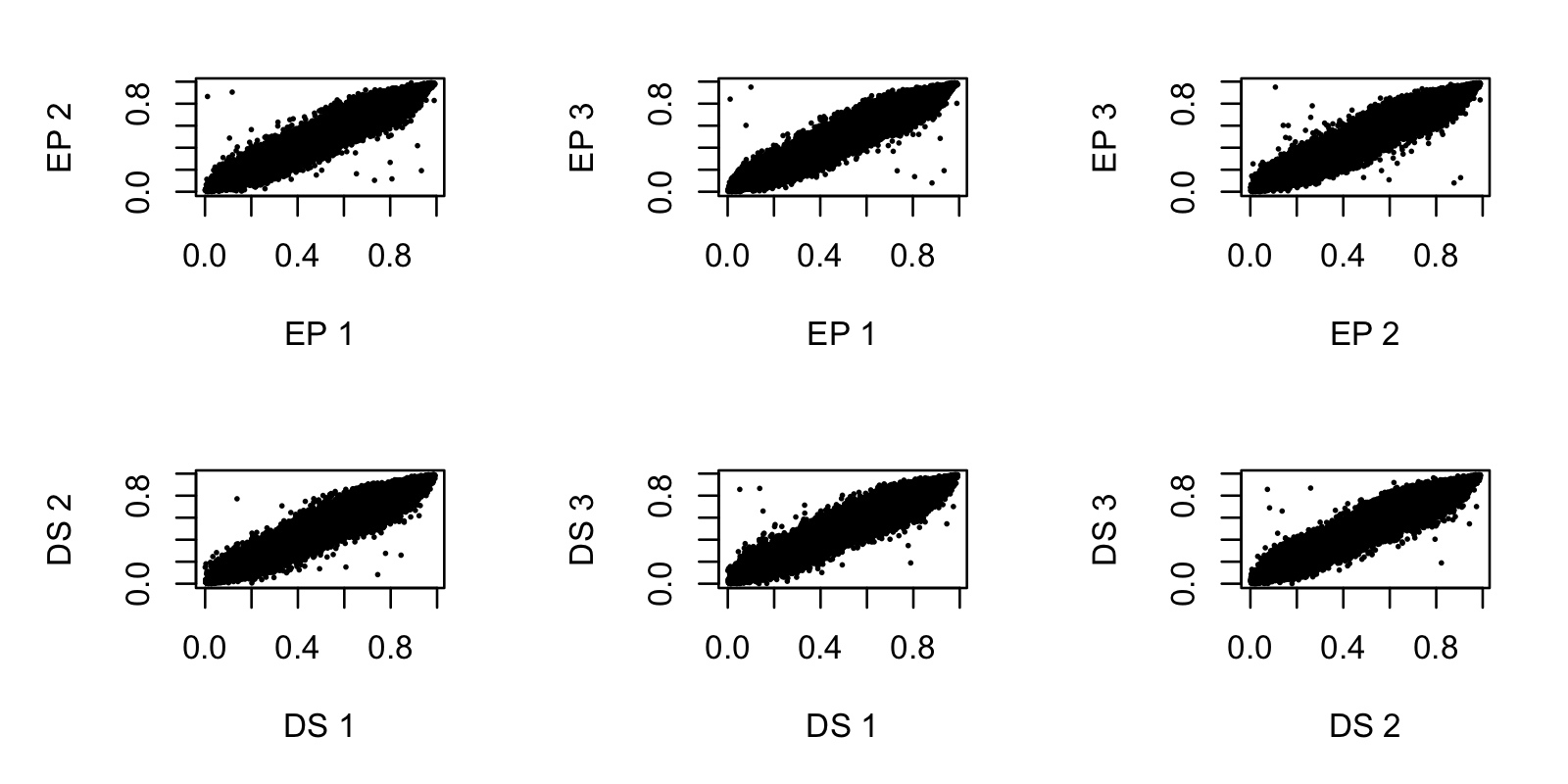


**Figure S3. Comparison of the** **methylation state between EP and DS technical replicates for experiment 1.** The methylation state as recorded by the beta value of the three triplicates in experiment 1 for both EP (EP1, EP2, EP3) and DS (DS1, DS2, DS3) for 401,915 probes analysed in the manuscript. EP 1 vs EP 2 (correlation: 0.996, p-value=0); EP 1 vs EP 3 (correlation: 0.996; p-value=0), EP 2 vs EP 3 (correlation: 0.995; p-value=0), DS 1 vs DS 2 (correlation: 0.995; p-value=0), DS 1 vs DS 3 (correlation: 0.995; p-value=0), DS 2 vs DS 3 (correlation: 0.995; p-value=0).

**
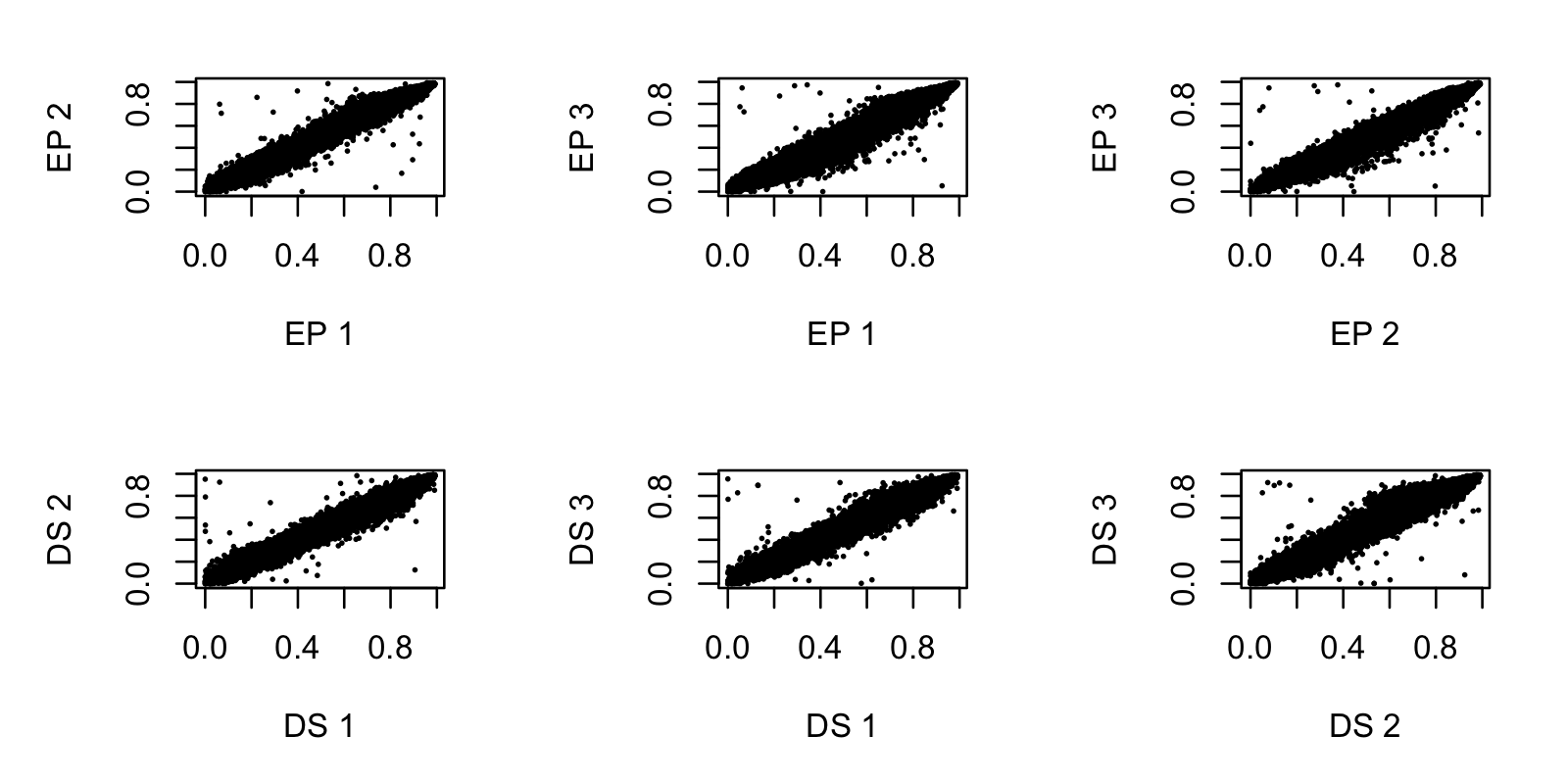
**

**Figure S4.** **Comparison of the** **methylation state between EP and DS technical replicates for experiment 2.** The methylation state as recorded by the beta value of the three triplicates in experiment 2 for both EP (EP1, EP2, EP3) and DS (DS1, DS2, DS3) for 401,915 probes analysed in the manuscript. EP 1 vs EP 2 (correlation: 0.999, p-value=0); EP 1 vs EP 3 (correlation: 0.998; p-value=0), EP 2 vs EP 3 (correlation: 0.999; p-value=0), DS 1 vs DS 2 (correlation: 0.998; p-value=0), DS 1 vs DS 3 (correlation: 0.998; p-value=0), DS 2 vs DS 3 (correlation: 0.998; p-value=0).

**
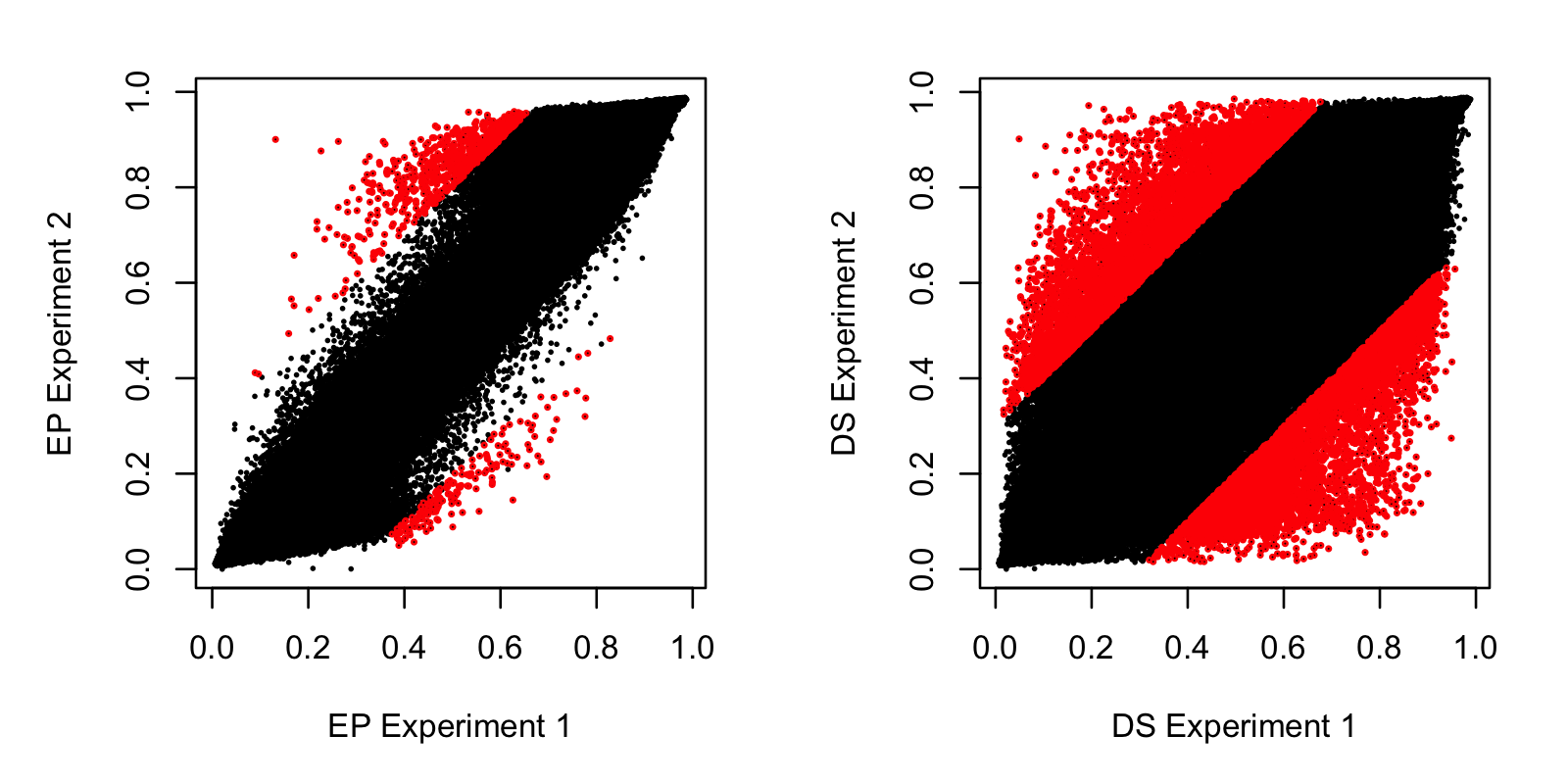
Figure S5. Comparison of average methylation between experiment 1 and 2 for EP and DS cells.** Scatterplot of the average methylation of the three triplicates at EP from experiment 1 (x-axis; left panel) compared to the triplicates at EP from experiment 2 (y-axis; left panel) for all probes (391,341) recorded in both experiment 1 and experiment 2 (black). Those found to be significantly differentially with genome wide corrected p-value < 0.01 and a beta value difference > 0.3 are shown in red (872). The right panel is a similar plot but for the average methylation of the triplicates at DS from experiment 1 (x-axis; right panel) against the triplicates at DS from experiment 2 (y-axis; right panel). Points highlighted in red are significantly different between the two experiments (11,568). The large number of sites significantly different at the DS stage in comparison to that of EP suggests that the EP methylation states are much more consistent across experiments.

**
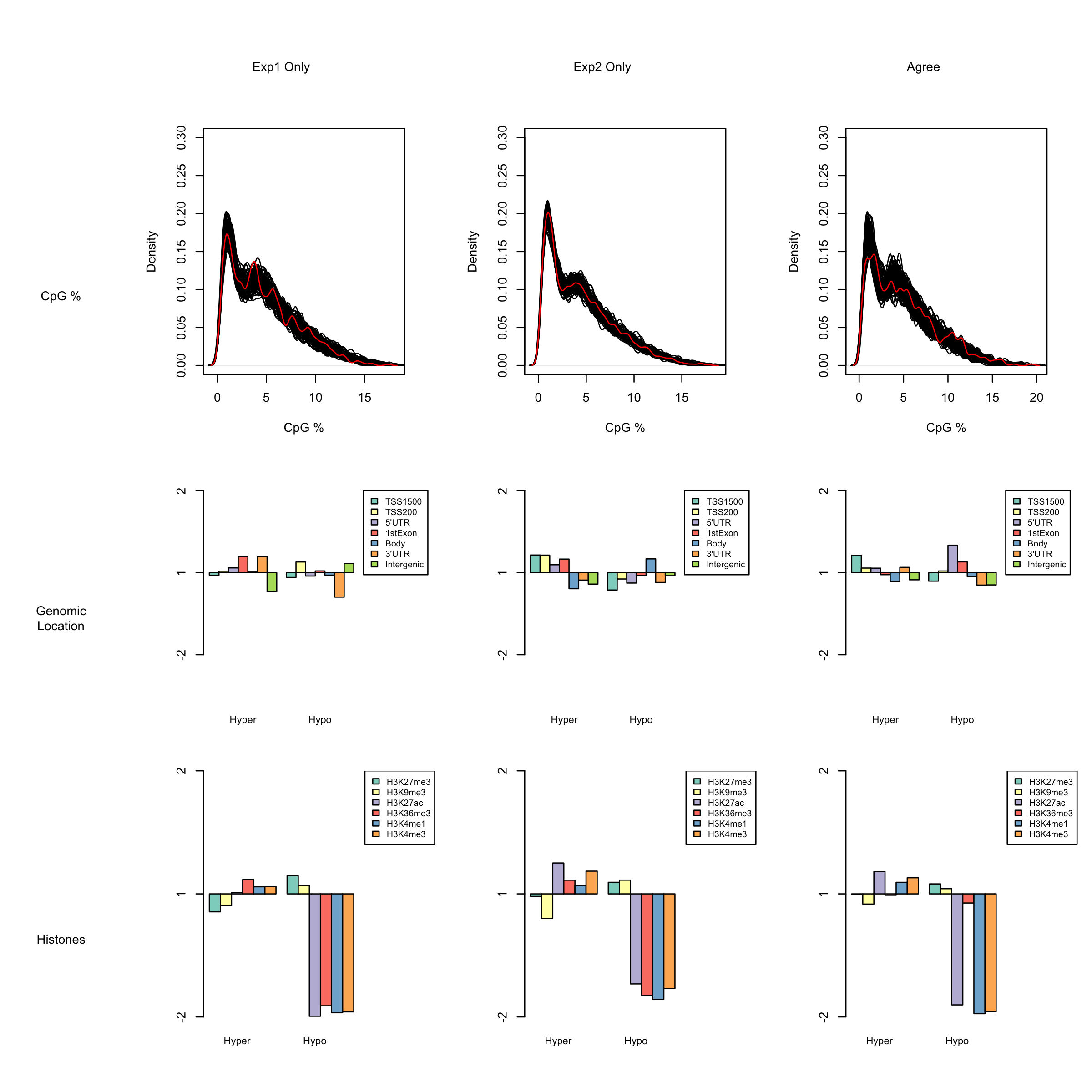
**

**Figure S6. Genomic feature analysis of senDMPs.** The columns represent the different groups of senDMPs with left column – experiment 1 only, middle column – experiment 2 only and right column – agree sites. Top row; shows CpG density profiles of senDMPs (red lines). In black are the background distributions derived by performing 1,000 different re-samplings using different random CpG sites. The second row shows relative enrichment/depletion of various annotated genomic features as defined in the Illumina450K annotation file. The bottom row shows relative enrichment/depletion of various histone modifications in HMECs previously profiled in the ENCODE project.

**
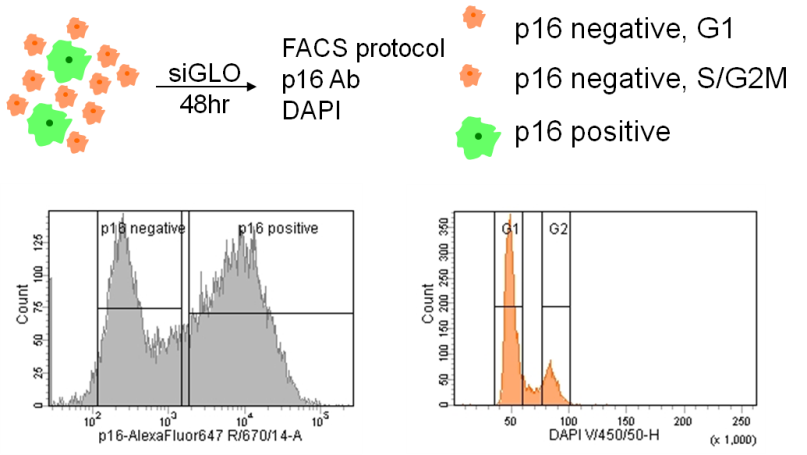
**

**Figure S7. The FACs sorting protocol.** Senescence (DS cells) is associated with a G1 cell cycle arrest, whereas all phases of the cell cycle are present during active proliferation (EP cells). Flow cytometry analysis was used to separate EP cells into G1-p16-ve, G2/S-p16-ve and G1-p16+ve fractions prior to Illumina450K array analysis.

**
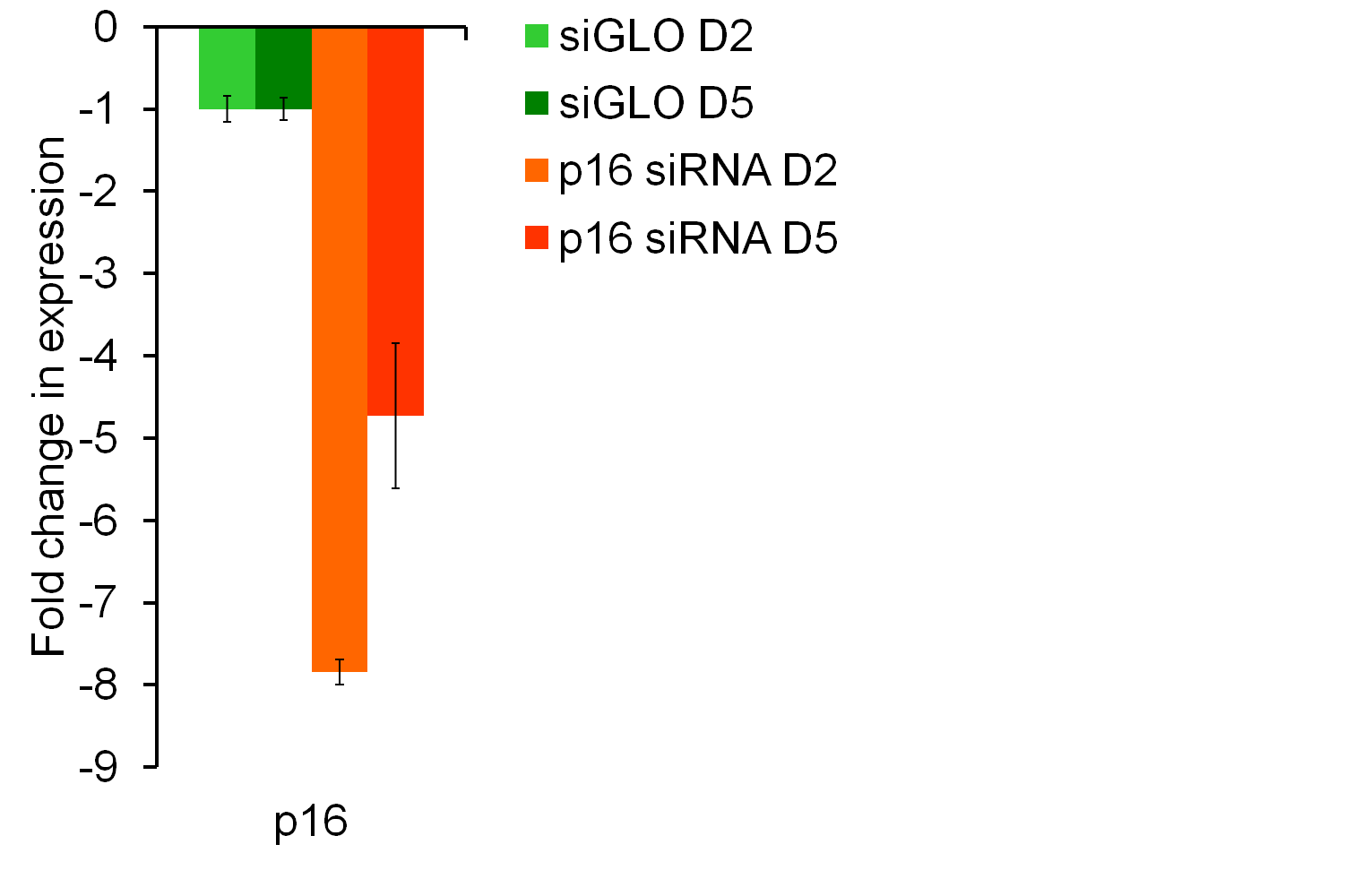
**

**Figure S8. Decreased expression of p16 following reversal.** Fold change in expression of p16 at day 2 and day 5 post-transfection of DS cells with p16 siRNA (orange) relative to DS cells transfected with siGLO (green) as control.


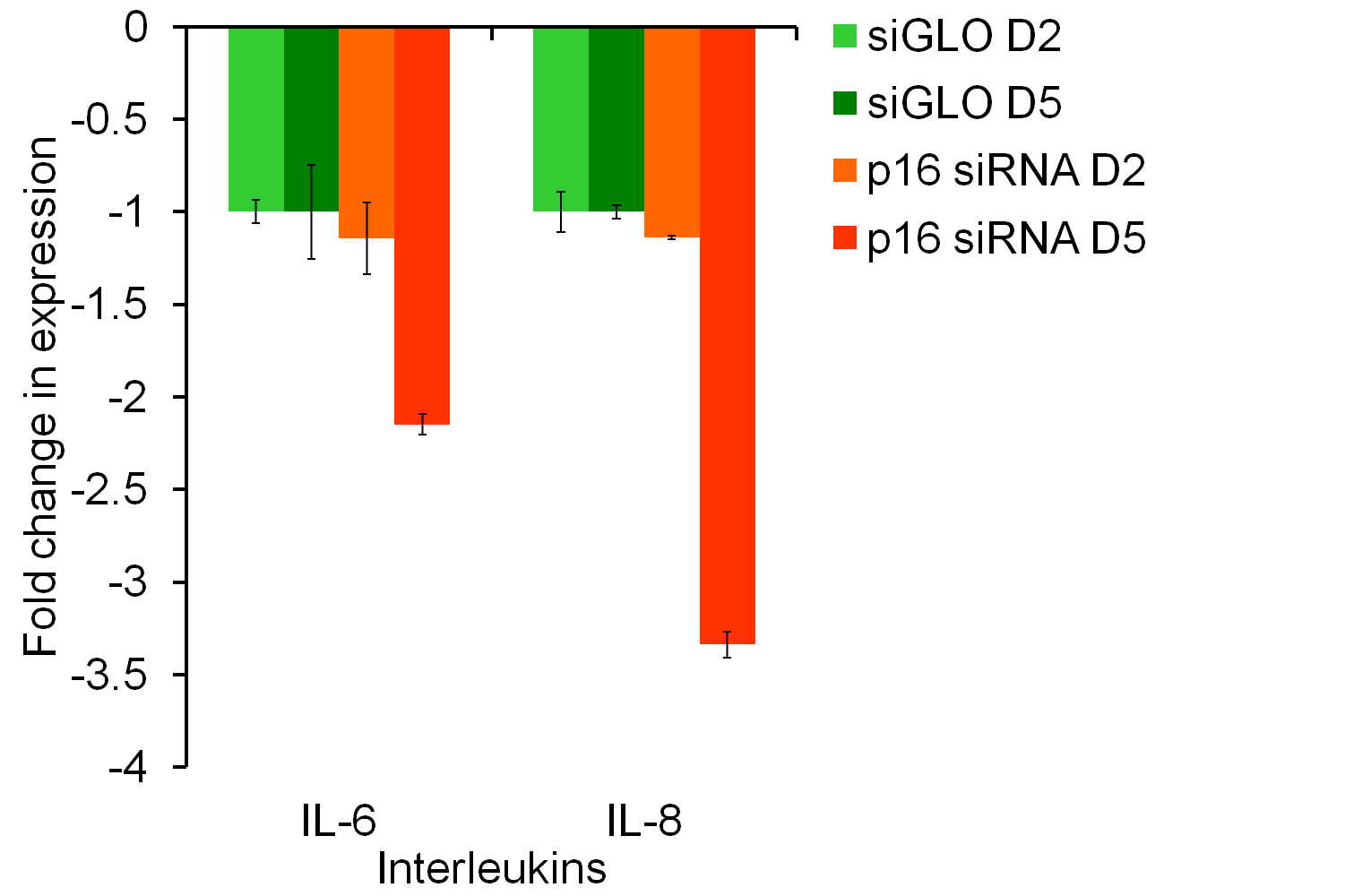


**Figure S9. Decreased expression of IL-6 and IL-8 following reversal.** Fold change in expression of IL-6 and IL-8 at day 2 and day 5 post-transfection of DS cells with p16 siRNA (orange) relative to DS cells transfected with siGLO (green) as control.

**
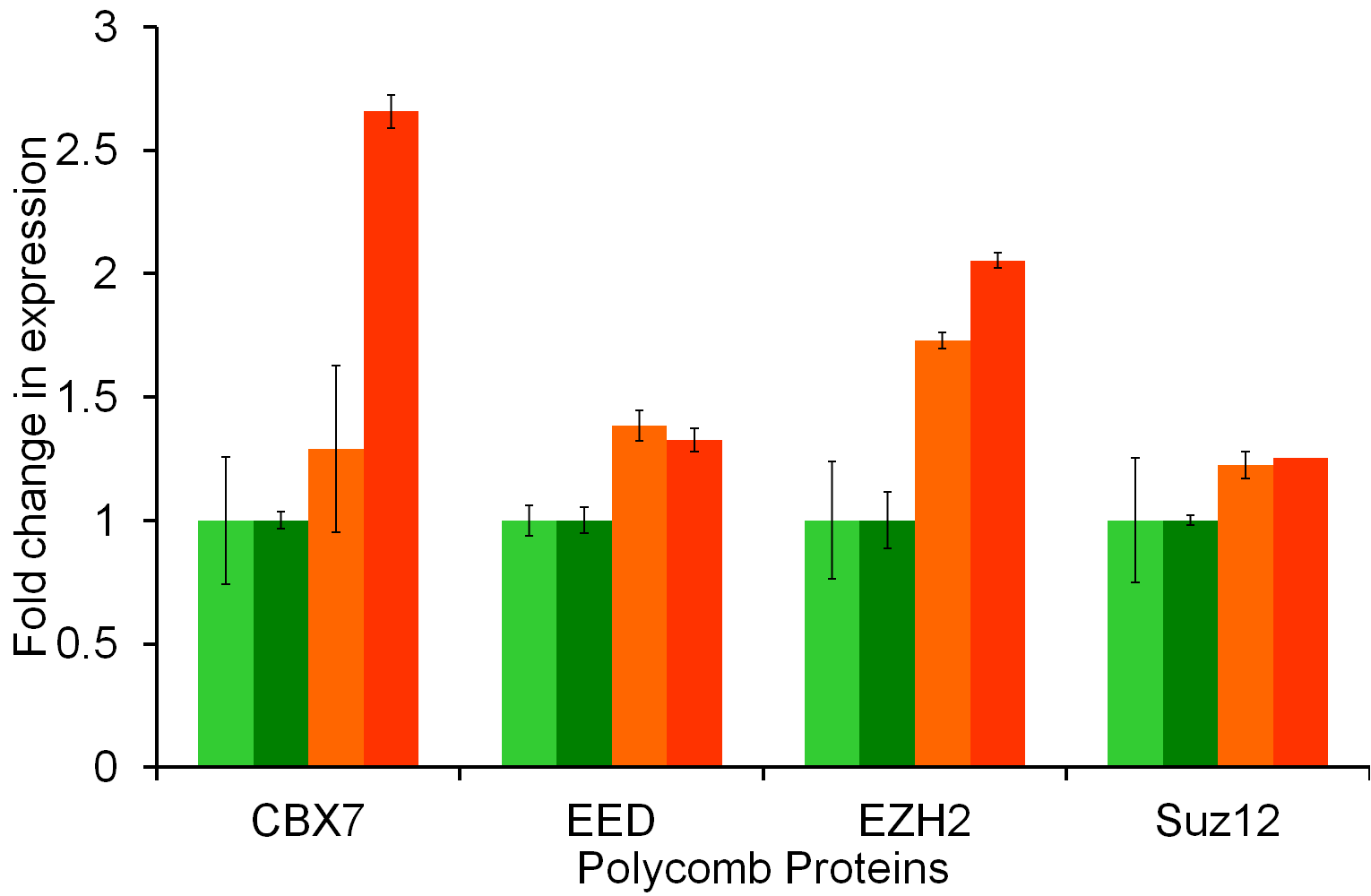
**

**Figure S10. Increased expression of Polycomb proteins following reversal.** Fold change in expression of Polycomb proteins CBX7, EED, EZH2 and Suz12 at day 2 and day 5 post-transfection of DS cells p16 siRNA (orange) relative to DS cells transfected with siGLO (green) as control.

**
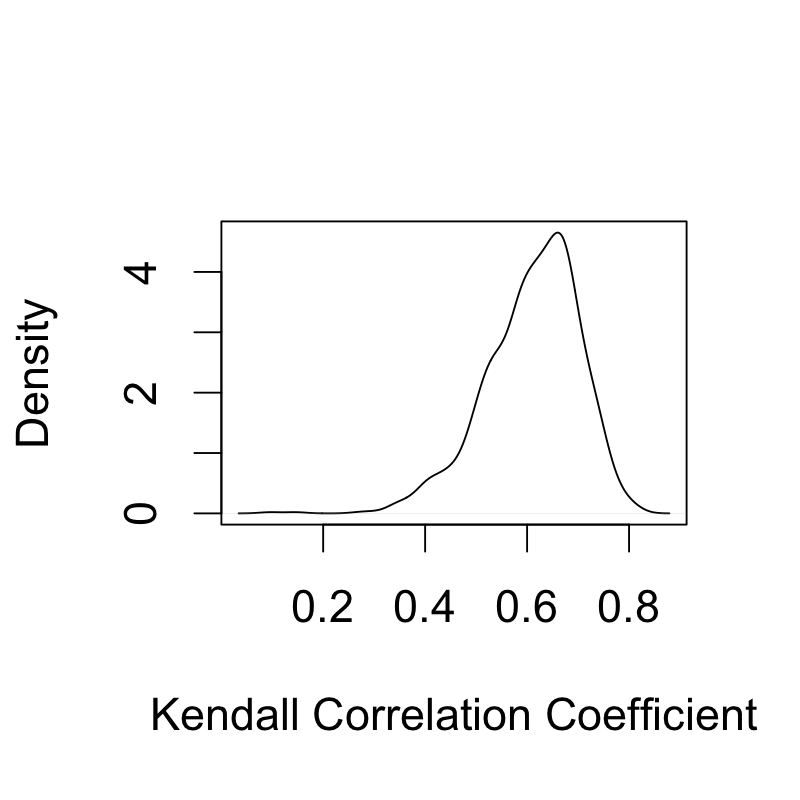
**

**Figure S11. Random permutation tests for hyper and hypo methylated Cpg sites.** A distribution of Kendall Correlation Coeffcients for a set of randomly chosen hyper and hypo methylated CpG sites. All random permutations show a positive correlation co-efficient and with an expected value of 0.61.

**
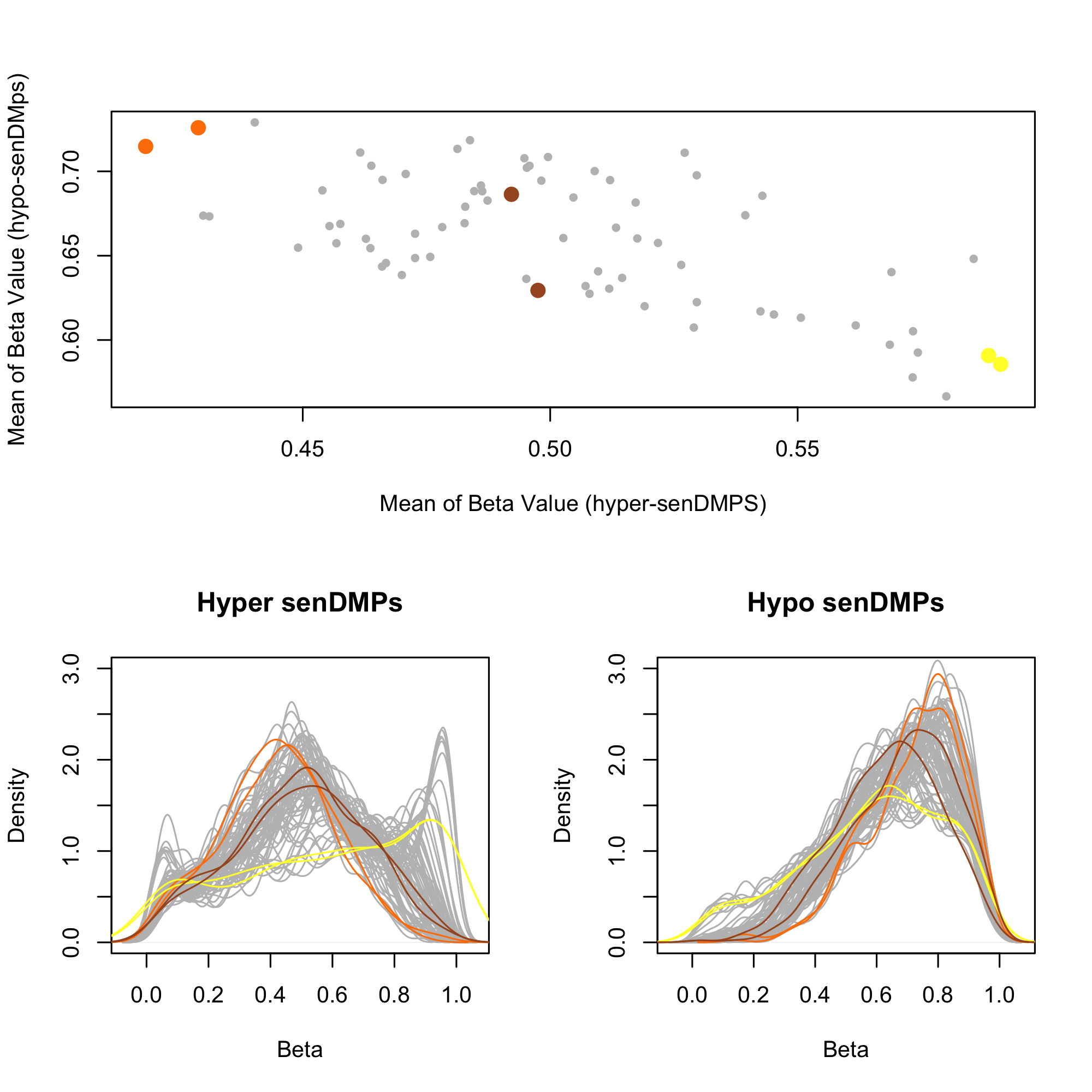
**

**Figure S12. Average beta values for senDMPs in normal breast tissue samples.** The top panel is a scatter plot of the average beta value of the hypermethylated-senDMPs (hyper-senDMPs [agree]) vs the average beta value of the hypomethylated-senDMPs (hypo-senDMPs [agree]) for the 73 normal breast tissue samples. The samples highlighted in yellow represent those with the lowest average beta vale of hyper-senDMPs, in orange represent the samples with the highest average beta value of hyper-senDMPs and in brown those within the middle of the highest and lowest. The bottom left panel shows the distribution of the beta values of the hyper-senDMPs for the 73 normal breast tissue in grey with the 6 samples as highlighted in the top plot (red lowest average value; blue highest average value and green middle value). The bottom right panel shows the distribution of beta values for hypo-senDMPs.

**
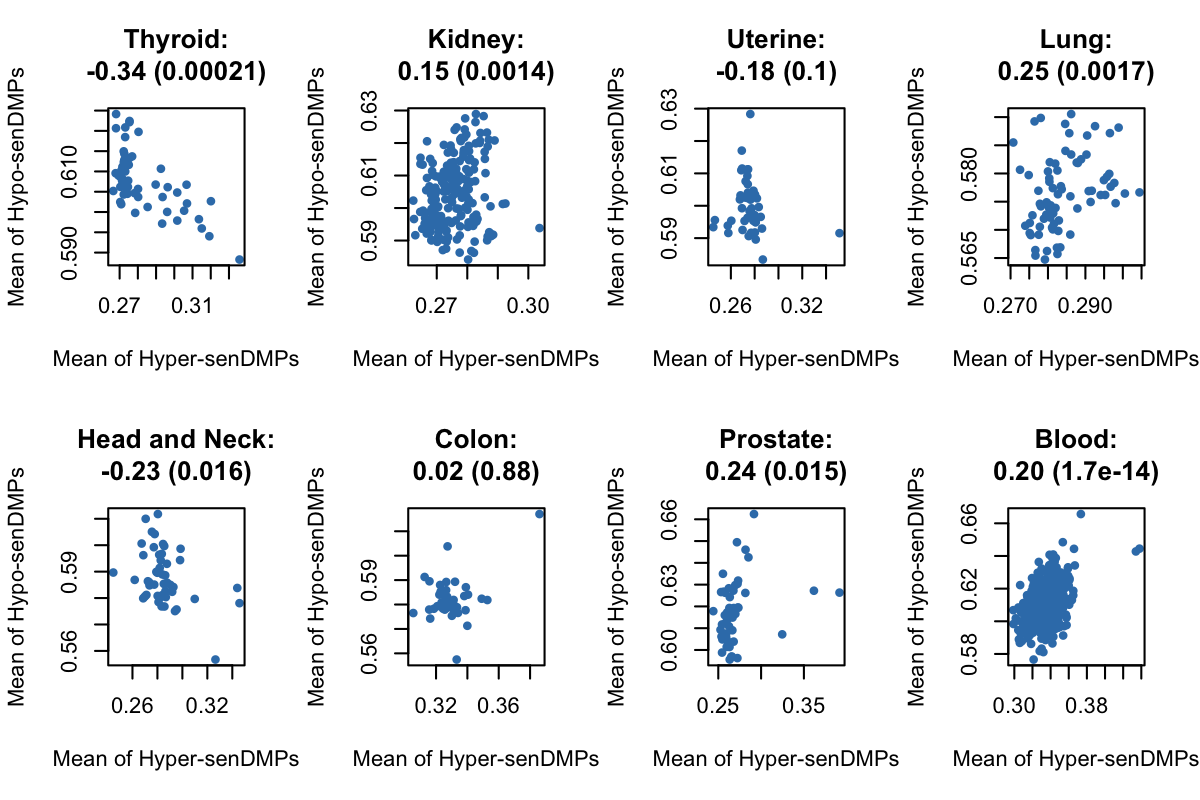
**

**Figure S13. Average beta values for experiment 1 only senDMPs in eight different tissues.** Scatter plots of various different tissues for the average beta value of hyper-senDMPs against the average beta value of hypo-senDMPs for each individual using the experiment 1 only senDMPs. The hyper (679) and hypo (290) senDMPs were those derived using our initial *in vitro* HMEC data. Tissue methylation data was extracted from the matched normal samples (e.g. normal samples from cancer patients) from the TCGA data set (See **Supplemental Methods -** **TCGA data**) excluding the Blood data, which was taken from Hannum *et al.,* 2013. The Kendall’s correlation co-efficient for each tissue is reported in the title of each panel and the associated p-value is shown in brackets.


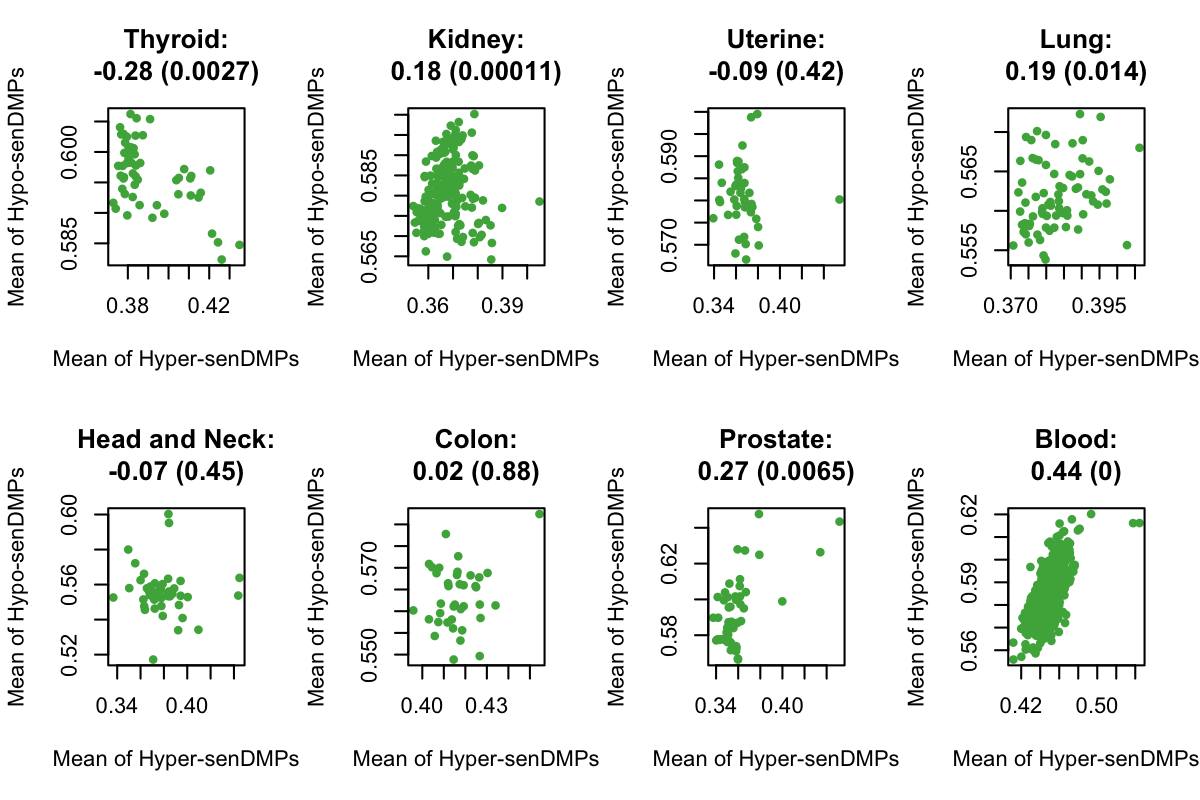


**Figure S14. Average beta values for experiment 2 only senDMPs in eight different tissues.** Scatter plots of various different tissues for the average beta value of hyper-senDMPs against the average beta value of hypo-senDMPs for each individual using the experiment 2 only senDMPs. The hyper (1,067) and hypo (740) senDMPs were those derived using our initial *in vitro* HMEC data. Tissue methylation data was extracted from the matched normal samples (e.g. normal samples from cancer patients) from the TCGA data set (See **Supplemental Methods -** **TCGA data**) excluding the Blood data, which was taken from Hannum *et al.,* 2013. The Kendall’s correlation co-efficient for each tissue is reported in the title of each panel and the associated p-value is shown in brackets.


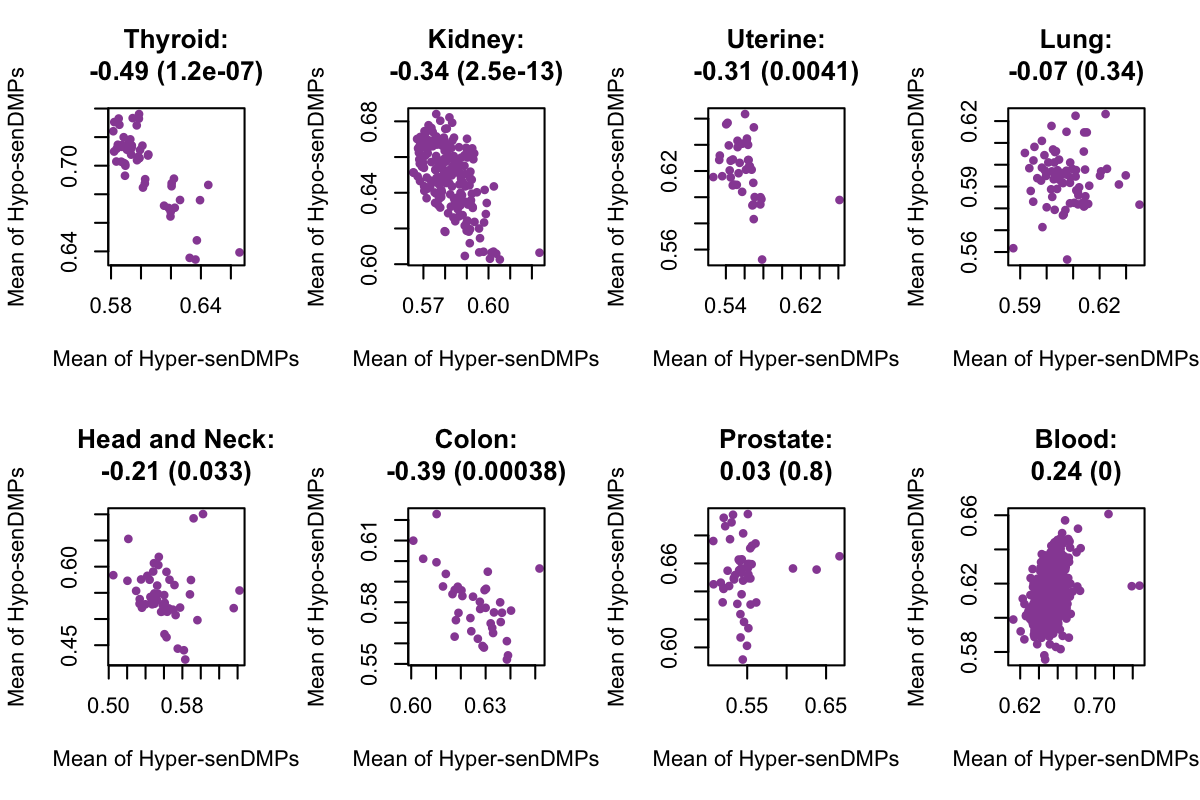


**Figure S15. Average beta values for agree only senDMPs in eight different tissues.** Scatter plots of various different tissues for the average beta value of hyper-senDMPs against the average beta value of hypo-senDMPs for each individual using the agree senDMPs. The hyper (313) and hypo (412) senDMPs were those derived using our initial *in vitro* HMEC data. Tissue methylation data was extracted from the matched normal samples (e.g. normal samples from cancer patients) from the TCGA data set (See **Supplemental Methods -** **TCGA data**) excluding the Blood data, which was taken from Hannum *et al.,* 2013. The Kendall’s correlation co-efficient for each tissue is reported in the title of each panel and the associated p-value is shown in brackets.

**
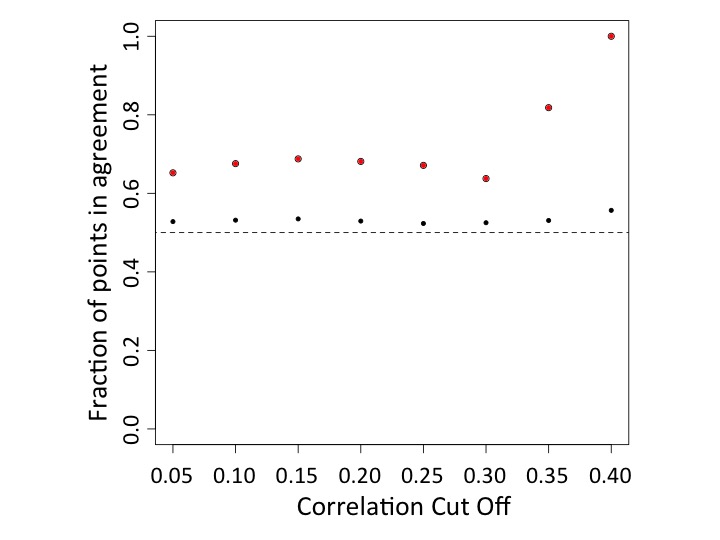
**

**Figure S16. Plot showing the fraction of hyper-senDMPs that showed a positive methylation and genotype correlation and hypo-senDMPs that showed a negative methylation genotype correlation (red) for different correlation cut offs.** In black is the average of 1000 permutations for randomly selected probes. There is a large increase in agreement using a correlation cut-off of 0.35 and 0.4 but this cut-off is too stringent and removes a large number of probes. We therefore settled on using a 0.15 correlation cut-off which had the next highest agreement but also did not remove too many probes.
